# Superior Communication of Positive Emotions Through Nonverbal Vocalisations Compared to Speech Prosody

**DOI:** 10.1007/s10919-021-00375-1

**Published:** 2021-07-24

**Authors:** Roza G. Kamiloğlu, George Boateng, Alisa Balabanova, Chuting Cao, Disa A. Sauter

**Affiliations:** 1 Department of Psychology, University of Amsterdam, REC G, Nieuwe Achtergracht 129 B, PO Box 15900, 1001 NK Amsterdam, The Netherlands; 2 Department of Management, Technology, and Economics, ETH Zürich, Zurich, Switzerland

**Keywords:** Acoustics, Machine learning, Nonverbal vocalizations, Positive emotions, Speech prosody, Voice

## Abstract

The human voice communicates emotion through two different types of vocalizations: nonverbal vocalizations (brief non-linguistic sounds like laughs) and speech prosody (tone of voice). Research examining recognizability of emotions from the voice has mostly focused on either nonverbal vocalizations or speech prosody, and included few categories of positive emotions. In two preregistered experiments, we compare human listeners’ (total *n* = 400) recognition performance for 22 positive emotions from nonverbal vocalizations (*n* = 880) to that from speech prosody (*n* = 880). The results show that listeners were more accurate in recognizing most positive emotions from nonverbal vocalizations compared to prosodic expressions. Furthermore, acoustic classification experiments with machine learning models demonstrated that positive emotions are expressed with more distinctive acoustic patterns for nonverbal vocalizations as compared to speech prosody. Overall, the results suggest that vocal expressions of positive emotions are communicated more successfully when expressed as nonverbal vocalizations compared to speech prosody.

**Supplementary Information:**

The online version contains supplementary material available at 10.1007/s10919-021-00375-1.

## Introduction

The human voice expresses a wealth of information, serving as an audible index of a person’s age, sex, identity, and emotional state (Kreiman & Sidtis, 2011). Among the features conveyed by the voice, an important element for everyday social interactions is the expression of emotions. Independently of semantic information (i.e., what is being said), the voice can express emotions via speech prosody (i.e., how it is being said), like speaking louder or softer. Emotion can also be expressed vocally via nonverbal vocalizations like screams and laughs. The voice can communicate discrete emotions such as anger, fear, and happiness to listeners via both nonverbal vocalizations and speech prosody (e.g., Banse & Scherer, 1996; Juslin & Laukka, 2001, 2003). However, it is poorly understood how the type of vocalization (nonverbal vocalizations vs speech prosody) influences listeners’ recognition of emotions. For example, is it easier to recognize amusement from a laugh as compared to a word spoken with amusement? Moreover, research to date has tended to include a limited number of positive emotions or to use a single positive emotion category, referred to as happiness or joy. In the present study, we examine recognizability of 22 positive emotions expressed via nonverbal vocalizations and speech prosody, and compare the accuracy levels between the two vocalization types.

### Nonverbal Vocalizations Versus Speech Prosody

Nonverbal vocalizations are brief non-speech expressions of emotions like laughs, moans, sighs, and grunts. They exclude linguistic interjections (e.g., “Surprise!”) that form part of semantic speech content (e.g., Ameka, 1992). Nonverbal vocalizations are similar to affect bursts (Scherer, 1994), but they do not include emblematic affect expressions like “yuck!” and they do not have to include a change in facial expressions. Nonverbal vocalizations are considered ‘pure’ expressions of emotions in the sense that they closely reflect physiological and autonomic changes (Scherer, 1986). Speech prosody, on the other hand, refers to suprasegmental attributes of spoken language, such as intonation, that can communicate emotions concurrently with semantic content (Juslin & Laukka, 2003; Scott et al., 2010). Acoustic features mainly related with rhythm and melodies in speech constitute the domain of prosody, which are sometimes referred to as paralinguistic features (Gibbon, 2017). It has been proposed that nonverbal vocalizations might be easier to understand than speech prosody (Scott et al., 2010). The production of emotional speech prosody is constrained because during speech production, there can be conflicts between the prosodic features associated with an emotional state and the ones used to denote linguistic information. For instance, changes in pitch levels signaling emotional information can conflict with changes relating to linguistic stress in a sentence or the rising pitch of a question. Such conflicts may create ambiguity in the speech prosody, resulting in less discriminable emotional information compared to nonverbal vocalizations. Nonverbal vocalizations, on the other hand, are largely unconstrained by linguistic structure (e.g., Pell et al., 2015; Scott, Sauter, & McGettigan, 2010). They are produced at the glottal/subglottal level with reduced volitional control of the vocal tract configurations (Trouvain, 2014). A lack of volitional control and linguistic constraints on nonverbal vocalizations leads to greater acoustic variability in nonverbal expression of emotions compared to prosodic expressions of emotions (e.g., Jessen & Kotz, 2011; Lima et al., 2013). Such flexibility might lead to the expression of emotions with higher discriminability in terms of acoustic structures.

Some emotions are typically expressed by means of nonverbal vocalizations and rarely vocalized via speech (Banse & Scherer, 1996). This proposal (yet untested) implies that recognizability advantage of nonverbal vocalizations over speech prosody might differ per emotion. For example, relief is typically expressed with sighs and amusement is expressed with laughs, with such vocalizations also occurring in other mammals, like rats (Panksepp & Burgdorf, 2003; Soltysik & Jelen, 2005). These emotions might have more distinctive acoustic configurations when expressed via nonverbal vocalizations compared to speech prosody, leading to higher recognizability. However, it is also a possibility that some emotions like feeling respected might not have any prototypical nonverbal vocalizations and might be preferred to be expressed through prosodic expressions. These emotions might have better differentiated acoustic profiles in speech prosody compared to nonverbal vocalizations.

Even though arguments for enhanced communication of emotions via nonverbal vocalizations as compared to speech prosody have been put forward, there is little research formally testing this notion. Studies conducted to date have found that negative emotions are recognized more accurately (Hawk et al., 2009; Lausen & Hammerschmidt, 2020; Sauter, 2007) and rapidly (Castiajo & Pinheiro, 2019; Pell et al., 2015; Schaerlaeken & Grandjean, 2018) from nonverbal vocalizations compared to speech prosody. For positive emotions, this perception advantage of nonverbal vocalizations has been tested for happiness/joy and pride, yet it is not established whether it generalizes to other positive emotions. In the present study, we compare human listeners’ recognition performance for nonverbal vocalizations and speech prosody for a wide range of different positive emotions.

Theorists have highlighted the need to differentiate between different positive emotions in vocal expressions for several decades. In an early review, Scherer (1986) lamented that such distinctions were rarely made in the literature and noted that it is therefore not clear what researchers refer to when they use the term ‘happiness,’ which makes it difficult to compare results between studies. Ekman (1992) suggested that ‘happiness’ should be replaced with several positive emotions and proposed that listeners might be able to differentiate these emotions from vocal expressions. The need to differentiate between positive emotions is further supported by an empirical study comparing more intense and less intense form of emotional vocalizations (Banse & Scherer, 1996). The two positive emotions, elation and happiness, were rarely confused with each other, suggesting that they might be expressions of two distinct positive emotions.

In recent years, researchers examining vocal communication of emotions are increasingly differentiating between distinct positive emotional states. There is empirical evidence showing that several positive emotions are expressed with vocal expressions characterized by distinct acoustic patterns and that they can be recognized by naïve listeners (see Kamiloğlu et al., 2020 for a review). However, studies to date have tended to include only a few categories of positive emotions and have focused on either nonverbal vocalizations or speech prosody. In the present study, we test whether listeners can recognize 22 different positive emotions from nonverbal vocalizations and speech prosody. We then test the robustness of these findings by conducting an identical experiment in a second cultural context.

## The Present Study

In the present study, we aim to compare recognition accuracy for nonverbal vocalizations to that from speech prosody for positive emotions. To do so, we first examined which of the 22 positive emotions could be recognized at better-than-chance levels for each type of vocalization. This allowed us to differentiate positive emotions that were not recognized from nonverbal vocalizations or speech prosody. We then tested the hypothesis that positive emotions are more accurately recognized when expressed as nonverbal vocalizations compared to speech prosody. We further sought to exploratorily examine recognition accuracy differences between the two vocalization types for each emotion. In order to be inclusive of a wide range of positive emotions, a total of 22 positive emotions that have been examined in the scientific literature were included: admiration, amae [presumption on others to be indulgent and accepting (Behrens, 2004)], amusement, awe, determination, elation, elevation, excitement, gratitude, hope, inspiration, interest, lust, moved, pride, relief, respected, schadenfreude, sensory pleasure, surprise, tenderness, and triumph (see Table 1 for definitions and examples).

**Table 1:** Positive emotions, accompanying definitions, and situational examples used in production of vocal expressions

Emotion	Definition	Situational example
Admiration	The feeling when you look up to someone who has excellent abilities or has accomplished impressive things. You have the urge to also achieve such things and to be more like this person	Betty is at work and her boss tells her she needs to give a presentation on the project she has been working on for 6 months. She has to deliver the talk in a few weeks. Until that point comes, she will be participating in many discussions on topics presented by her other colleagues. During one of those meetings her co-worker, Angela, gives a talk herself about a different project. Betty admires Angela for her presentation skills. Betty is truly impressed with the quality of the talk and the way Angela dealt with the questions afterwards. Betty looks up to how confident Angela seemed during the talk and truly views Angela as a role model.
Amae	The feeling when you think about or interact with a person or entity to whose love, care, and protection you want to submit yourself	Mark has been dating Clare for a year now. They have become very close and Mark feels he truly loves Clare. When he is with her, he feels that she really cares for him and he likes this feeling. It makes him feel light-hearted and he wants to indulge into this loving sensation as much as possible. When he sees Clare, he wants her to embrace him and hold him in her arms as he feels secure, loved, and even a bit spoiled.
Amusement	The feeling when you encounter something silly, ironic, witty, or absurd, which makes you feel entertained. You have the urge to be playful and share the joke with others	Gill was feeling a bit stressed. As she was walking towards the cafeteria, she saw her friend John walking down the stairs. Gill waved at him and John smiled and hurried down, so that he could have his coffee with Gill. However, as he was walking, he slipped on the last step and spilled his strawberry smoothie all over his face and white shirt. Gill couldn’t help herself, but finding this situation extremely funny. Her clumsy friend was alright, but now all covered in the pinky smoothie, he joined Gill to share her amusement at the situation. She could barely breathe and her eyes have watered!
Awe	The feeling when you encounter something that is greater or more powerful than yourself. You feel a sense of overwhelming positivity and need a moment to adjust	Ross went on a hike with his friend Nick. They were hiking for hours until they reached the top of the mountain. From there they could see a vast view—from the gorgeous lake separating the hills to the far-away golden clouds. The sun was setting in the distance, painting everything in bright beautiful colors. Ross and Nick were mesmerized at this view. They felt awe, the feeling of mind-blowing beauty of nature. What an extraordinary experience!
Determination	The feeling when you are working on a demanding problem or task that you believe you can handle. You feel stimulated to accomplish it	Susan likes jigsaw puzzles. She has just bought a new one with 1500 pieces. It seems very difficult to put together, because there are many similar shapes, and the colors are faint. It will be a difficult task to put it together, but Susan is very motivated to finish it. She is making a plan for solving the puzzle. Susan is determined and she believes that she has what it takes to complete the jigsaw puzzle.
Elation	The feeling when something very good just happened to you. You have the urge to celebrate and share it with others	Peter has been struggling with money for some time now. He doesn’t believe in luck, but decides to buy a lottery scratch card as they are on promotion in the supermarket. When he gets home, Peter scratches out the card and discovers that he has won 10 000 euro. How great is that, Peter can barely believe his luck. Peter is so happy, he feels completely euphoric as he rushes to share the great news with his wife.
Elevation	The feeling when you witness someone’s noble or good deed. You have the urge to praise this person and to also do good yourself	On the way to work, Kaley notices a crowd gathering near one of Amsterdam’s many bridges. She rushes to see what is happening and is terrified to discover that a little boy has fallen into the canal. Before Kaley has time to think, a young woman jumps in the water with all her clothes on, and attempts to save the child. The boy is safely rescued and the woman who saved him makes sure to find the child’s parents. Noticing this noble act, Kaley feels elevated, having an optimistic feeling about humanity. She believes everyone is capable of conducting a virtuous act and feels extremely generous herself.
Excitement	The feeling when you expect that something good or nice will happen to you. You cannot stop thinking about this	Sam is planning his big trip to Australia. He has been saving up for a year to afford the vacation and now is finalizing the last few details. Sam is really looking forward to all the surfing he will do, to visiting all the famous, beautiful nature sites and, most of all, to seeing his childhood friend Ben, who has been living in Sydney for five years. Sam is very excited about the trip, he feels energized and thrilled just thinking about it. It’s going to be awesome!
Gratitude	The feeling when you think that someone has gone out of their way to do something good or nice for you. You have the urge to do something good back and to get closer to this person	Christen was a big Jules Verne fan as a child. She has read all of his books and especially liked “An adventure to the center of the world”. For Christen’s birthday, her friend Maike gave her an original first print of the book. Christen was so thankful and happy. She couldn’t believe it! Maike must have gone through a lot of trouble to find that edition—it was very rare, and also quite expensive. Christen was so grateful for the gift, she was feeling full of gratitude and appreciation towards Maike. She wished she could do something as nice for her one day.
Hope	The feeling when you believe that something you want to happen has a possibility of happening. You keep thinking about how good it would be if it actually happened	Keith is planning his next trip with his childhood friends. He doesn’t see them very often, so this trip is really exciting for all of them. They plan on doing some hiking in the Alps while backpacking and sleeping under the stars. Now the only thing left is for the weather to be on their side. A rainstorm can easily ruin the whole trip as the boys are not well prepared for such conditions. Keith really hopes the weather will be good. He wishes he could order it to be all sunny and warm. Regardless, he is optimistic that everything will go as planned and just desires to have a good old time with his friends.
Inspiration	The feeling when you suddenly get a new idea or insight, or see the world in a different light. You have the urge to express or actualize this new insight	Sanne has always liked arts. She really enjoys painting and writing short stories. However, recently she has been lacking ideas for her stories. When some school friends invited her to an art exhibition, she took the opportunity to go along. Little did she know how inspired she would feel after seeing some of the art work! The exhibition was a grand success and definitely gave her plenty of input for her creative work. She felt motivated to go back home and start writing, feeling creative and full of ideas.
Interest	The feeling when you encounter something new and relevant that you do not immediately understand. You have the urge to find out more about it	Tom recently returned from his trip to Italy, where he managed to visit many of the famous cities: Milano, Rome, Venice, Florence, and Pisa. What really sparked his curiosity was the leaning tower of Pisa. During the trip, the tour guide gave a detailed story of how the tower was built. Since Tom studied architecture, this story really stuck with him. He later talked to the guide again and wanted to learn even more. Tom was absolutely fascinated by it and wanted to learn more about its history and its future. How was it possible for it to be still standing after so many years of slowly, but surely, tilting itself to the side? Tom was so interested in it that he read several books and searched for as many articles as he could find.
Lust	The feeling when you think about or interact with someone that you find sexually attractive. You have the urge to be physically close and have sexual relations with this person	John came back home after a very long day at work. He was a bit tired, but when he opened the door, he was happily surprised by his girlfriend waiting for him in a set of revealing red lingerie. She gave him a quick smile and came near him to kiss him passionately. She took his hand inviting him towards their bedroom. John was feeling very turned on and wanted to appreciate his lovely girlfriend’s body. He was full of excitement. John really craved that passionate moment of desire and seduction.
Moved	The feeling when you encounter something very beautiful, meaningful, or bittersweet. Tears well up in your eyes and you feel overcome with warm feelings	Vicky was recently on the airport waiting for her flight to Malaga. She had a few hours until she had to go to her gate. As she walked around the airport, she found herself near the arrivals. What caught her attention was a woman with a small child dressed in camouflage waiting with a sign (“Welcome home, daddy”). And soon enough Vicky saw a tall handsome soldier appearing from behind the doors. He ran towards the woman and hugged her and the child so strongly that they appeared suspended in the air as he was lifting them up in a moment of happiness. Vicky felt so moved and emotional at the view. She could see the happy tears of the reunited family and she felt touched.
Pride	The feeling when you (or someone close to you) possess or have accomplished something that other people find praiseworthy	This year Luuk is being promoted to a senior position in his job. He has been working for a few years in the company and is very happy to receive the recognition he deserves. On the day of the promotion his boss tells him how lucky he is to have him in his team. Luuk felt as if this was one of his greatest achievements so far and was touched by his boss’s words.
Relief	The feeling when an unpleasant experience is finally over, or when you find out that something you had dreaded has not happened or will not happen. You can finally take your mind off it	The news announced that a huge tropical storm is coming and people from the coast sides are quickly evacuated. Kate is one of them. She doesn’t manage to grab much—only her passport and her beloved dog. Her house is just next to the sea, so she is very scared of how the storm might damage it. Kate learns that the storm also comes with a huge tidal wave and although she is glad she is safe and sound, she can’t keep her mind off her house and the damage the storm will do to it and to all her belongings. When the storm is finally over, she comes back home to find her beloved home untouched. As it turned out, the storm changed direction just before hitting the shore, so little damage was done to Kate’s neighborhood. She was relieved that it was all over. Kate heaved a sigh and moved on with her life happy that everything was in place.
Respected	The feeling when you're valued and recognized, treated politely. You are deemed worthy listening and understanding by others	Paul asked Jamie for some advice about buying his first own flat. Jamie is happy to help his friend and after advising him, the two friends have a long discussion. Paul listens carefully to what Jamie has to say and asks him to elaborate, while making noting many important details. Jamie feels respected and looked up to. His friend really values his opinion and listens to his advice.
Schadenfreude	The feeling when something bad happens to another person. You enjoy this because you dislike the person, because you think the person deserves it, or because it is somehow good for you	Martin is walking down the street and enjoying the sunshine when suddenly, out of nowhere, a man appears, and starts offending him. Martin tries to pass by the stranger, but then the man blocks his way and continues with the insults. Since Martin doesn’t want to get into trouble, he turns and walks the other away. Hearing a loud noise, he turns around to see the stranger fallen down in a pound. The stranger is not hurt, but all wet and covered with mud. Martin secretly smiles to himself, as he is happy the guy got what he deserved. Might be just karma.
Sensory Pleasure	The feeling when something happens that pleases your senses	Meisie was walking around in the living room when she smelled the flavor of something being baked in the kitchen. She rushed towards the door and noticed her flatmate, Sasha whisking some cream and then chopping some fruits. She gave Meisie a spoon to try, and oh, my what a delicious frosting it was! Meisie felt great enjoyment and genuine pleasure. Yum!
Surprise	The feeling when you realize something good or nice just happened, which you did not expect. You need a moment to take in the good news	Roy got promoted to an important and long-desired position at work today. He hurried to tell his girlfriend, Sarah, and was already planning on taking her out to celebrate. He came home with flowers, but as soon as he opened the door he saw a whole group of his closest friends along with Sarah screaming: “Congrats, Roy!”. He was so positively surprised! Roy couldn’t have imagined a better celebration after years of work! He was pleasantly taken aback and felt amazed at how wonderfully his girlfriend managed to surprise him.
Tenderness	The feeling when you encounter someone or something that seems cute, vulnerable, or childlike in appearance or behavior. You have the urge to nurture and care for this person or thing	Kim was walking in the park on a lovely summer afternoon. He noticed a small pup running towards him and almost falling on the side—it still had not learned how to run on the uneven surface. It was wagging its tail and happily collapsed at Kim’s feet. Kim gently stroked the puppy and it looked up at him with its big eyes and wagged its tail energetically. Kim felt tenderness towards the small doggie, he felt fond of it and wished he could take it home and take good care of it.
Triumph	The feeling of release and a great joy, after a successful ending of a struggle or contest	Lily was competing for the ice skating team in her home town. She reached the national finals and then went to the European championship. She was very nervous presenting her country and at a few times wanted to bail. However, when the time came for her performance everything went smoothly and she gave the best performance of her lifetime. When announcing the winners she couldn’t believe that it was she who took the golden medal! What a triumph! She felt brilliant after such a victory and was full of joy.

In Experiment 1, naïve Dutch listeners were asked to complete a forced-choice emotion categorization task for vocal expressions produced by native Dutch speakers. Experiment 2 was a replication of Experiment 1 in which naïve Chinese listeners completed an identical forced-choice emotion categorization task with vocalizations of 22 positive emotions produced by native Chinese Mandarin speakers. The hypotheses, methods, and data analysis plan for both experiments were pre-registered on the Open Science Framework (https://osf.io/6c8v3/?view_only=) before data collection was commenced.

In order to compare acoustic patterns of positive emotions expressed via nonverbal vocalizations to that of speech prosody, we conducted an acoustic analysis. Machine learning models were used to classify the nonverbal vocalizations and speech prosody stimuli from Experiment 1 and 2 based on their acoustic features. We hypothesized that acoustic classification accuracy of positive emotions would be higher for nonverbal vocalizations compared to speech prosody.

## Experiment 1: Dutch Listeners’ Recognition of Positive Emotions from Dutch Vocalizations

In Experiment 1, we first examine whether naïve Dutch listeners would be able to recognize 22 positive emotions from vocal expressions produced by native Dutch speakers at levels significantly above chance. We then test whether recognition of positive emotions is better from nonverbal vocalizations as compared to speech prosody overall, and provide a breakdown of the results per emotion.

### Method

#### Participants

We estimated the sample size through data simulation. A generalized linear mixed model was constructed to test whether participants would recognize 22 positive emotions at better-than-chance levels, with the dependent variable being a binary response (correct or incorrect). Positive emotion with 22 factors was set as a fixed effect. Participant and vocalization IDs were entered as random factors accounting for participant and speaker variability. Chance level was set to 1/8, which is the chance of selecting the correct emotion category by random guessing in an 8-way forced-choice task. We defined logit for the reference probability of 1/8, which was entered in the model as an offset term. The simulations indicated that using a sample size of *N* = 200 would ensure that the experiment would be well powered (80%) for testing whether recognition performance would be at better-than-chance levels. Simulations were run in R (version 1.1.383, www.r-project.org) using lme4 package (Bates, Mächler, Bolker, & Walker, 2015). The simulation script is provided in Supplementary Materials Script 1S.

Two hundred native Dutch speakers (105 women, 92 men, 2 other, 1 preferred not to say; *M*_*age*_ = 21.75, *SD*_*age*_ = 3.82, range = 18–40 years old) with no (self-reported) hearing impairments were recruited via the University of Amsterdam, Department of Psychology’s research pool, and by advertisements posted on Facebook. Participation in the study was compensated with course credit or monetary reward.

#### Materials and Procedure

##### Stimuli

Posed vocal expressions of positive emotions were recorded at the University of Amsterdam’s psychology laboratory. The walls of the laboratory were covered with high quality acoustic fabric to prevent echoes. Individuals whose native language was Dutch and who had never been diagnosed or treated for any voice, speech, hearing, or language disorder were considered eligible for participation in the study. Twenty participants (10 women, 10 men; *M*_*age*_ = 22.42, *SD*_*age*_ = 2.64, range = 20–31 years old) were invited to the lab to record vocalizations.

Upon arriving at the lab, participants were seated in front of a lab computer, which displayed each emotion term in turn, together with its definition. Participants were then asked by the experimenter to describe the emotion in their own words to ensure that they understood the definition correctly. If needed, they were provided examples. Then, they read a situational example and were asked to imagine the situation as vividly as possible. They were then asked to produce a vocal expression of the corresponding emotion. The target emotions, accompanying definitions, and situational examples can be found in Table 1.

Participants were positioned approximately 30 cm from the microphone. They produced both nonverbal vocalizations and speech prosody for each of the 22 positive emotions. When producing nonverbal vocalizations, participants were asked to avoid actual words (e.g.,” “no,” “yes”) and vocalizations with conventionalized semantic meanings (e.g., “yuck,” “ouch”). For speech prosody, speakers were asked to produce the semantically neutral word “zeshonderd zevenenveertig” (from Dutch: six hundred forty-seven) in a way that expressed the target emotion. We chose to use a neutral word to make sure that vocal emotions would be communicated solely in terms of prosodic cues. Participants were instructed to avoid inserting any additional sounds such as laughs or sighs into their speech (e.g., Hawk et al., 2009). Each type of vocalization (nonverbal vocalizations and speech prosody) constituted a separate block. The order of the two blocks and the order of emotions in each block were randomized across participants. Participants were allowed to produce multiple vocal expressions for a given emotion. If they did, they were asked to choose the expression they thought best depicted the emotion they were trying to express. All stimuli were recorded using a high-quality microphone (Sennheiser MKE 600) and a Tascam DR-100 MK3 recorder.

In total, 880 vocalizations were collected. Average duration was 1.30 s (*SD* = 0.56) for nonverbal vocalizations, and 2.28 s (*SD* = 0.44) for speech prosody. All of the vocalizations were used in the recognition experiment without any preselection. Before the recognition experiment, recordings were digitalized at a 44 kHz sampling rate (16 bit, mono) and normalized for peak amplitude using AudaCity software (version 2.2.2, https://www.audacityteam.org). A representative vocalization for each positive emotion and vocalization type can be listened from https://emotionwaves.github.io/dutch22/.

##### Experimental Procedure

The recognition study was run online using the Qualtrics (Provo, UT) survey tool. Before the experiment, participants were instructed to complete the experiment in a silent environment and to use headphones. After being informed about the general procedure and giving informed consent, they were provided with the definitions of 22 positive emotions (see Table 1). After reading the definitions, participants completed two practice trials, each of which played an emotional vocalization that was not included in the main experiment (taken from www.findsounds.com). After listening to each vocalization, they were asked to select the emotion they thought the individual was expressing, choosing from eight response options. During the practice trials, participants were asked to adjust to a comfortable sound level and to keep it constant for the rest of the study. After the practice trials, participants were presented with two screening questions. One of these played a bird sound and the other a car horn. On these trials, participants were asked to indicate what they heard, with “bird sound” and “car horn” as response options. These questions were used to make sure that participants were paying attention and listening to the stimuli. Participants who failed one or both of the screening questions were not able to continue to the main experiment.

After the practice and screening questions, participants were assigned to one of fourteen conditions, half of which were speech prosody, and the other half nonverbal vocalizations. In each condition, three stimuli from each emotion category (e.g., three nonverbal vocalizations expressing admiration) were presented. Each participant thus completed 66 trials (22 emotion × 3 vocalization) in total. This way, each of the 880 stimuli were judged by at least one participant. After hearing each vocalization, participants were asked to make a forced-choice emotion categorization judgment, selecting from eight emotion categories (“Select the positive emotion you think the individual was expressing”). These response options included the target category (i.e., the emotion category of the stimulus on that given trial), and seven nontarget categories (emotion categories randomly selected from the remaining 21 positive emotions). Across all participants, all target response categories were paired with all nontarget response categories. For instance, across trials that included admiration vocalization as stimuli, the nontarget response options included all of the other 21 emotion categories for some participant(s). The presentation order of stimuli was randomized for each participant, and the response options were presented in a randomized order on each trial. There was no time constraint on completing each trial, and participants were able to replay each stimulus as many times as needed to make a judgment.

#### Statistical Analysis

Statistical analysis and outlier detection were done based on the preregistered analysis plan. Before the analysis, data were checked for participants with exceptionally low performance, defined as 3 SD or more below the mean in terms of overall recognition performance. None of the participants met this criterion, and so all were retained in the analyses.

We constructed a generalized linear mixed model (GLMM) to analyze whether listeners were able to categorize positive emotions at better-than-chance levels for nonverbal vocalizations and speech prosody. GLMM was used because it allows for fixed effects to be defined in addition to taking advantage of the computation of random effects. Positive emotion was set as a fixed effect. Participant and vocalization IDs were entered as random factors to account for participant and speaker variability. Chance level was set to 1/8, which is the probability of selecting the correct emotion category by random guessing. We defined logit for the reference probability of 1/8, which was entered into the model as an offset term. The dependent variable was a binary response (i.e., correct or incorrect response):

glmer (response ~ offset(logit(1/8)) + PositiveEmotion + (1|ParticipantnID) + (1|VocalizationID), family = binomial)

To test our prediction that participants would recognize emotions from nonverbal vocalizations better than from speech prosody, we constructed a second GLMM. In this model, type of vocalization was set as fixed effect, and similarly to the previous model, participant and vocalization IDs were entered as random factors:

glmer (response ~ offset(logit(1/8)) + Type + (1|VocalizationID) + (1|ParticipantID), family = binomial)

All analyses were performed in R (version 1.1.383, www.r-project.org) using the lme4 package (Bates, Mächler, Bolker, & Walker, 2015).

### Results

**Fig. 1:**
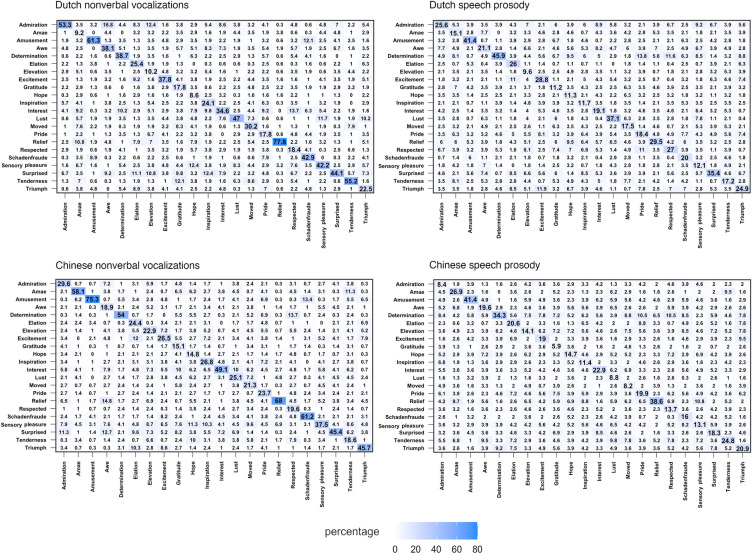
Heatmap of confusion matrices (%) for positive emotions categorization data. The x-axes represent stimuli and the y-axes indicate responses

Confusion matrices for average recognition percentages for nonverbal vocalizations and speech prosody for each emotion are shown in Fig. 1. Recognition performance compared to the chance level per emotion is shown in Table 2.

**Table 2:** GLMM model comparing emotion recognition performance to chance level (1/8)

Fixed Effects	Dutch nonverbal vocalizations		Dutch speech prosody		Chinese nonverbal vocalizations		Chinese speech prosody	
	*Z*	*p* ( >|*z*|)	Z	*p* ( >|*z*|)	Z	*p* ( >|*z*|)	Z	*p* ( >|*z*|)
Admiration	6.336	** < 0.001**	3.135	**0.002**	2.982	**0.003**	− 1.807	0.071
Amae	− 1.934	0.053	− 0.239	0.811	5.651	** < 0.001**	1.361	0.174
Amusement	6.576	** < 0.001**	3.092	**0.002**	11.08	** < 0.001**	5.125	** < 0.001**
Awe	3.541	** < 0.001**	1.642	0.101	1.553	0.12	2.271	**0.023**
Determination	8.726	** < 0.001**	8.721	** < 0.001**	6.586	** < 0.001**	4.775	** < 0.001**
Elation	0.83	0.407	1.838	0.066	2.937	**0.003**	0.999	0.318
Elevation	− 1.254	0.21	− 1.449	0.147	2.792	**0.005**	0.031	0.975
Excitement	4.522	** < 0.001**	339.1	** < 0.001**	2.246	**0.025**	0.74	0.459
Gratitude	0.68	0.497	− 1.231	0.218	− 1.069	0.285	− 2.164	0.03
Hope	− 1.988	0.047	− 1.313	0.189	− 1.407	0.159	0.675	0.5
Inspiration	2.384	**0.017**	− 1.208	0.227	3.159	**0.002**	− 0.99	0.322
Interest	5.061	** < 0.001**	1.615	0.106	7.307	** < 0.001**	3.311	** < 0.001**
Lust	4.762	** < 0.001**	2.133	**0.033**	2.345	**0.019**	− 1.698	0.089
Moved	2.605	**0.009**	41.42	** < 0.001**	1.513	0.13	− 1.976	0.048
Pride	0.94	0.347	0.491	0.624	2.961	**0.003**	2.792	**0.005**
Relief	7.795	** < 0.001**	2.548	**0.012**	8.641	** < 0.001**	4.658	** < 0.001**
Respected	0.916	0.36	3.124	**0.002**	− 1.183	0.237	− 0.899	0.369
Schadenfreude	6.1	** < 0.001**	− 0.275	0.784	8.841	** < 0.001**	− 0.2	− 0.841
Sensory Pleasure	6.1	** < 0.001**	− 0.956	0.339	7.976	** < 0.001**	− 31.11	< 0.001
Surprise	8.933	** < 0.001**	7.752	** < 0.001**	5.456	** < 0.001**	− 0.211	0.833
Tenderness	6.472	** < 0.001**	0.833	0.405	0.14	0.888	1.956	0.051
Triumph	0.907	0.365	2.462	**0.012**	4.889	** < 0.001**	2.398	**0.017**

Bold indicates performance accuracy at significantly better-than-chance levels

Fourteen positive emotions were recognized at better-than-chance level when expressed as nonverbal vocalizations. These emotions, ordered based on coefficients for fixed effects in log-odd scale, were relief (*Est.* = 4.274, *SE* = 0.331), amusement (*Est* = 2.484, *SE* = 0.378), tenderness (*Est.* = 2.194, *SE* = 0.339), admiration (*Est.* = 2.099, *SE* = 0.331), lust (*Est.* = 1.919, *SE* = 0.376), surprise (*Est.* = 1.671, *SE* = 0.187), sensory pleasure (*Est.* = 1.642, *SE* = 0.269), schadenfreude (*Est.* = 1.642, *SE* = 0.269), determination (*Est.* = 1.451, *SE* = 0.166), excitement (*Est.* = 1.308, *SE* = 1.308), interest (*Est.* = 1.233, *SE* = 0.244), awe (*Est.* = 1.170, *SE* = 0.330), being moved (*Est.* = 0.821, *SE* = 0.315), and inspiration (*Est.* = 0.628, *SE* = 0.244). These findings demonstrate that many positive emotions are recognizable from nonverbal vocalizations for naïve listeners.

**Fig. 2:**
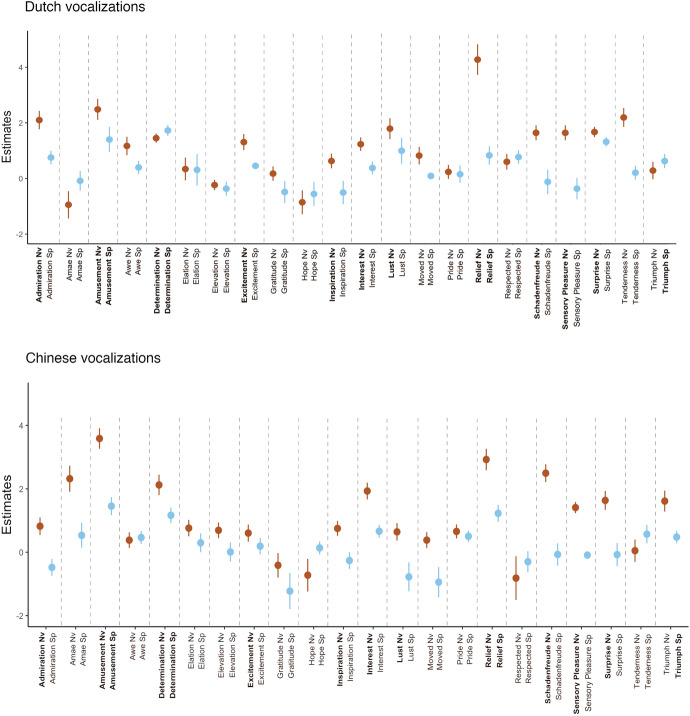
Forest plots of estimates of the GLMMs. The x-axes represent estimates of the fixed effects as log-odds with standard error bars. Larger standard error bar indicates higher uncertainty about coefficient point estimates. Zero estimate indicates no recognition. Positive emotions recognized better than the chance level for the corresponding vocalization type in both cultural contexts are marked in bold

For speech prosody, 10 positive emotions were recognized better than would be expected by chance. These emotions, ordered by coefficient size for fixed effects in log-odd scale, were determination (*Est.* = 1.725, *SE* = 0.198), amusement (*Est.* = 1.401, *SE* = 0.453), surprise (*Est.* = 1.671, *SE* = 0.187), lust (*Est.* = 0.997, *SE* = 0.467), relief (*Est.* = 0.830, *SE* = 0.326), being respected (*Est.* = 0.771, *SE* = 0.247), admiration (*Est.* = 0.753, *SE* = 0.240), triumph (*Est.* = 0.626, *SE* = 0.254), excitement (*Est.* = 0.454, *SE* = 0.001), and being moved (*Est.* = 0.091, *SE* = 0.002). These results suggest that some positive emotions can be recognized from speech prosody. Figure 2 illustrates the estimates from the GLMM models. Full details of the GLMMs are provided in the Supplementary Materials, Tables S1 and S2.

**Fig. 3:**
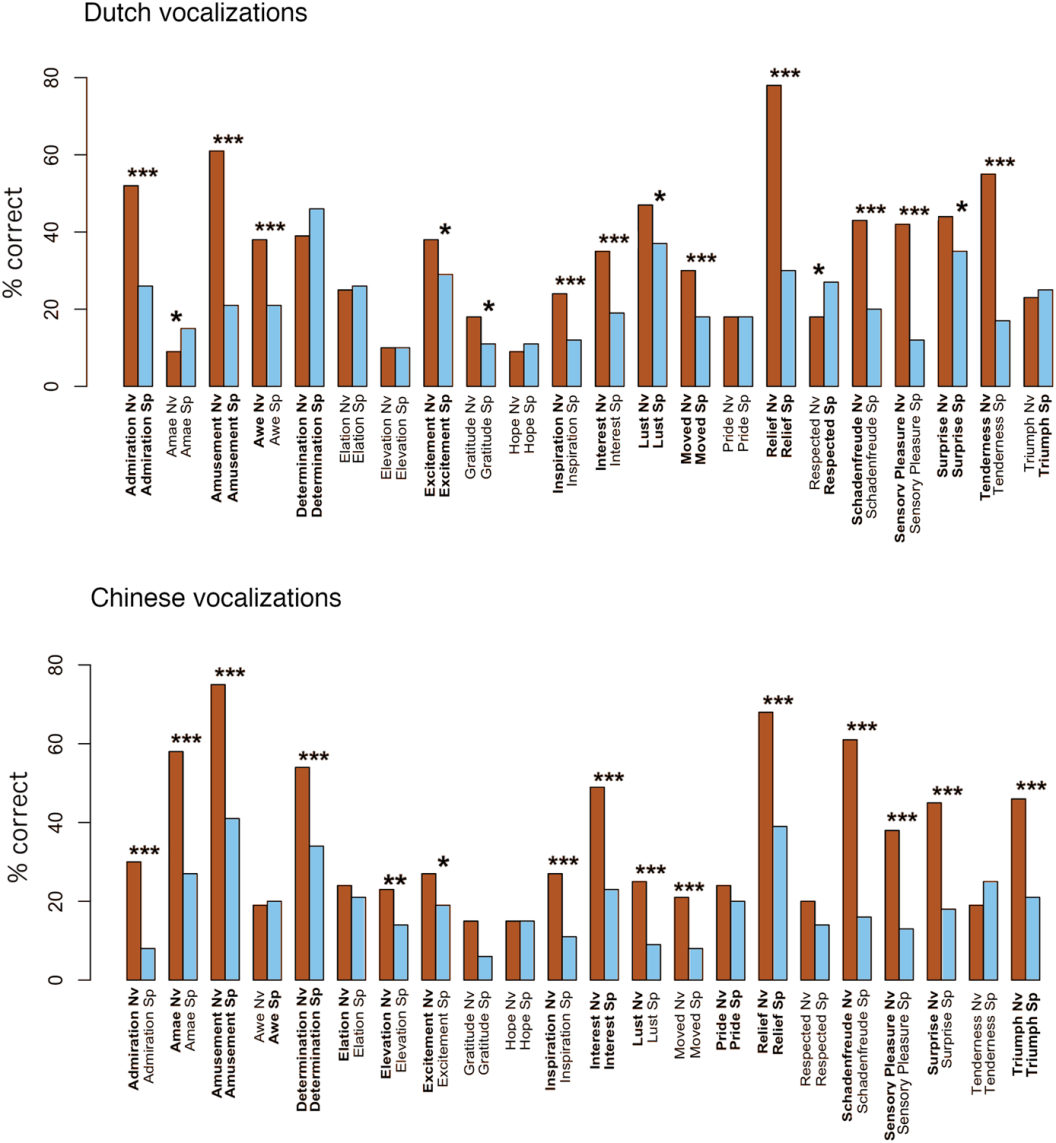
Correct responses in percentages per emotion across vocalization types for Dutch and Chinese vocalizations. Bold text indicates recognition with above chance level accuracy tested with GLMM models. Significance levels comparing vocalization types: *** < 0.001, ** < 0.01, * < 0.05. Nv = Nonverbal vocalizations, Sp = Speech prosody

We hypothesized that positive emotions would be recognized with higher accuracy rates from nonverbal vocalizations compared to speech prosody. The results showed that participants categorized nonverbal vocalizations of positive emotions significantly better than speech prosody overall (GLMM: *z* = − 8,599, *p* < 0.001). When performance accuracy was compared for each emotion separately, 14 positive emotions were recognized with significantly better accuracy from nonverbal vocalizations. However, two emotions were recognized better from speech prosody than nonverbal vocalizations: amae and feeling respected (see Table 3). However, not all of these emotions were recognized better-than-chance level for both expressions of nonverbal vocalizations and speech prosody, suggesting that some emotions can only be recognized from some one kind of vocal expression. Awe, inspiration, interest, schadenfreude, sensory pleasure, and tenderness were recognized better than expected by chance only when expressed via nonverbal vocalizations. In contrast, feeling respected was accurately recognized only from the prosodic expressions. This might be an emotion that is expressed by differentiable prosodic configurations in speech, but lacking a unique nonverbal vocalization. Even though amae was recognized better when expressed via speech prosody as compared to nonverbal vocalizations, it was not recognized from either vocalization type at above chance levels. However, in many cases, positive emotions were recognized from both nonverbal vocalizations and speech prosody at better-than-chance levels, with recognition being higher for nonverbal vocalizations compared to speech prosody. Figure 3a illustrates the comparisons of accurate responses across vocalization types per emotion. Random effects in GLMM models are summarised in Supplementary Materials, Table S3.

**Table 3:** GLMM models comparing emotion recognition performance across vocalization types per emotion

Fixed effects	Dutch				Chinese			
	Est	*SE*	*Z*	*p* ( >|*z*|)	Est	*SE*	*Z*	*p* ( >|*z*|)
**Admiration**	− 1.350	0.222	− 6.075	< 0.001	− 1.608	0.278	− 5.783	< 0.001
Amae	0.615	0.293	2.100	0.036	− 1.728	0.258	− 6.694	< 0.001
**Amusement**	− 1.131	0.231	− 4.894	< 0.001	− 1.975	0.277	− 7.136	< 0.001
Awe	− 1.l09	0.270	− 4.046	< 0.001	0.016	0.219	0.075	0.941
Determination	0.301	0.171	1.757	0.079	− 0.923	0.212	− 4.345	< 0.001
Elation	0.021	0.214	0.101	0.920	− 0.346	0.210	− 1.648	0.099
Elevation	− 0.078	0.275	− 0.281	0.778	− 0.719	0.220	− 3.276	0.001
**Excitement**	− 0.454	0.202	− 2.246	0.025	− 0.554	0.226	− 2.450	0.014
**Gratitude**	− 0.502	0.247	− 2.034	0.042	− 1.036	0.377	− 2.749	0.006
Hope	0.335	0.289	1.160	0.246	0.007	0.266	0.027	0.978
**Inspiration**	− 1.107	0.278	− 3.981	< 0.001	− 1.123	0.241	− 4.654	< 0.001
**Interest**	− 0.876	0.200	− 4.371	< 0.001	− 1.335	0.215	− 6.206	< 0.001
**Lust**	− 0.505	0.202	− 2.501	0.012	− 1.467	0.304	− 4.821	< 0.001
**Moved**	− 0.805	0.210	− 3.824	< 0.001	− 1.215	0.305	− 3.986	< 0.001
Pride	0.011	0.246	0.045	0.964	− 0.213	0.203	− 1.048	0.295
**Relief**	− 2.905	0.348	− 8.341	< 0.001	− 1.695	0.222	− 7.611	< 0.001
Respected	0.501	0.203	2.470	0.014	− 0.531	0.331	− 1.601	0.109
**Schadenfreude**	− 1.422	0.249	− 5.715	< 0.001	− 2.426	0.278	− 8.733	< 0.001
**Sensory Pleasure**	− 2.042	0.284	− 7.190	< 0.001	− 1.370	0.217	− 6.318	< 0.001
**Surprise**	− 0.349	0.175	− 1.990	0.047	− 1.615	0.233	− 6.936	< 0.001
Tenderness	− 2.098	0.256	− 9.196	< 0.001	0.379	0.213	1.779	0.075
Triumph	0.137	0.220	0.624	0.533	− 1.350	0.200	− 6.740	< 0.001

Bold indicates better emotion recognition performance for nonverbal vocalizations as compared to speech prosody in both cultural contexts. No emotion was consistently better recognized from speech prosody across the two contexts

Taken together, the results from Experiment 1 showed that 16 of the 22 positive emotions were recognized better than would be expected by chance level by naïve Dutch listeners from the vocal expressions of Dutch speakers. Moreover, 14 positive emotions were recognized better when expressed via nonverbal vocalizations compared to prosodic expressions, indicating superior recognition of most positive emotions from nonverbal vocalizations. As compared to speech prosody, nonverbal vocalizations of most positive emotions might have relatively distinctive acoustic profiles that are highly differentiated from those of other emotions, leading to higher recognition scores.

## Experiment 2: Chinese Listeners’ Recognition of Positive Emotions from Chinese Vocalizations

Experiment 1 was conducted in a Dutch cultural context. In order to evaluate the robustness of the findings, we sought to repeat Experiment 1 in a distant cultural context. Languages are characterized by prosodic conventions, which might shape the communication of emotions via speech prosody. Choosing distant cultures with different prosodic conventions allows us to interrogate the robustness of the findings, making it unlikely that the same prosodic conventions shape the communication of positive emotions in our study. In Experiment 2, we test whether (1) Chinese listeners can recognize 22 positive emotions from nonverbal expressions and speech prosody from stimuli produced by Chinese individuals; and (2) whether positive emotions would better recognized from nonverbal vocalizations of as compared to prosodic expressions also in a Chinese cultural context.

### Method

#### Participants

Sample size was determined in the same way as Experiment 1. Two hundred native Chinese Mandarin speakers (109 women, 90 men, 1 prefer not to say; *M*_*age*_ = 27.51, *SD*_*age*_ = 4.50, range = 19–35 years old) with no (self-reported) hearing impairments were recruited via a Chinese online data collection platform, https://www.wjx.cn. Participation in the study was compensated with a monetary reward.

#### Materials and Procedure

##### Stimuli

Posed vocal expressions of positive emotions in Chinese Mandarin were recorded at the University of Amsterdam’s psychology laboratory, using the same procedure as the recordings of the Dutch vocalizations (see Experiment 1, Stimuli). Eligibility criteria for participating in the recordings were: (1) being a native Chinese Mandarin speaker, (2) having been in the Netherlands for no more than 3 months by the time of the recording, (3) having lived in China until the age of 18, and (4) never having lived outside of China for more than 2 years. Based on these criteria, twenty participants (10 women, 10 men; *M*_*age*_ = 23, *SD*_*age*_ = 2.63, range = 19–31 years old) were invited to the laboratory to record vocalizations. Participants reported never having been diagnosed or treated for any voice, speech, hearing, or language disorder.

The experimenter was a Chinese Mandarin native speaker, and the entire recording procedure was in Chinese Mandarin. The target emotions, accompanying definitions, and situational examples (given in Table 1), as well as the neutral phrase used for recordings of speech prosody (“六百四十七” from Chinese Mandarin: six hundred forty-seven), were provided in Chinese Mandarin. All 880 recorded vocalizations were used as stimuli in Experiment 2. Average duration as 1.25 s (*SD* = 0.64) for nonverbal vocalizations, and 1.64 s (*SD* = 0.45) for speech prosody. An example of vocalizations for each positive emotion and vocalization type is available from https://emotionwaves.github.io/chinese22/.

##### Experimental procedure

The experimental procedure was the same as in Experiment 1, except that the stimuli were from the Chinese Mandarin recordings.

#### Statistical Analysis

For data analysis and outlier detection, the preregistered plan was followed. Before data analysis, data were checked for participants with 3 SD or more below the mean on overall recognition performance. Based on this criterion, one participant’s data were excluded from the analysis. The statistical analyses were identical to those employed in Experiment 1.

### Results

Confusion matrices for average recognition percentages for nonverbal vocalizations and speech prosody are shown in Fig. 1. Comparisons of recognition performance to chance level per positive emotion for nonverbal vocalizations and speech prosody can be found in Table 2.

Sixteen positive emotions were recognized at better-than-chance level from nonverbal vocalizations. In the order of coefficient size in log-odd scale, these emotions were amusement (*Est.* = 3.587, *SE* = 0.324), relief (*Est.* = 2.924, *SE* = 0.338), schadenfreude (*Est.* = 2.494, *SE* = 0.282), amae (*Est.* = 2.319, *SE* = 0.410), determination (*Est.* = 2.123, *SE* = 0.322), interest (*Est.* = 1.931, *SE* = 0.264), surprise (*Est.* = 1.635, *SE* = 0.300), triumph (*Est.* = 1.613, *SE* = 0.330), sensory pleasure (*Est.* = 1.408, *SE* = 0.177), admiration (*Est.* = 0.821, *SE* = 0.275), elation (*Est.* = 0.762, *SE* = 0.259), inspiration (*Est.* = 0.751, *SE* = 0.238), elevation (*Est.* = 0.692, *SE* = 0.248), pride (*Est.* = 0.656, *SE* = 0.222), lust (*Est.* = 0.643, *SE* = 0.274), and excitement (*Est.* = 0.604, *SE* = 0.269). These findings show that nonverbal vocalizations are highly effective means of conveying many positive emotions.

In contrast, only seven positive emotions were recognized better than would be expected by chance from speech prosody. These emotions in the order of coefficients in the log-odd scale were amusement (*Est.* = 1.453, *SE* = 0.284), relief (*Est.* = 1.227, *SE* = 0.263), determination (*Est.* = 1.165, *SE* = 0.244), interest (*Est.* = 0.662, *SE* = 0.200), pride (*Est.* = 0.503, *SE* = 0.180), triumph (*Est.* = 0.479, *SE* = 0.200), and awe (*Est.* = 0.465, *SE* = 0.205). These results suggest that prosodic expressions are not very effective in conveying positive emotions, with recognizability highly dependent on the emotion expressed. Estimates from the GLMM models are visualised in Fig. 2. Full details of the GLMMs are provided in the Supplementary Materials, Tables S1 and S2.

As in the Dutch cultural context, we sought to test the hypothesis that positive emotions would be more accurately recognized from nonverbal vocalizations than from speech prosody. As predicted, participants categorized nonverbal vocalizations of positive emotions better than speech prosody overall (GLMM: *z* = − 10.69, *p* < 0.001). Next, we compared performance accuracy across vocalization types for each emotion, showing that 16 positive emotions were recognized with better accuracy from nonverbal vocalizations. None of the emotions was more accurately recognized from speech prosody (see Table 3). It is worth noting that not all of the 16 emotions that were recognized better from nonverbal vocalizations than speech prosody were recognized above chance levels for both kinds of expressions (see Fig. 3b). Admiration, amae, elation, elevation, excitement, inspiration, lust, surprise, sensory pleasure, and triumph were recognized at better-than-chance levels only when expressed as nonverbal vocalizations. These emotions might thus be expressed with unique nonverbal vocalizations, while they are not clearly communicated via speech prosody cues. These results suggest that recognizability of some positive emotions depends on the vocalization type through which the emotion is expressed. Summary of random effects in GLMM models can be found in Supplementary Materials, Table S3.

Experiment 2 revealed that naïve Chinese listeners recognized 17 out of 22 positive emotions better than expected by chance from vocal expressions of native Chinese Mandarin speakers. Moreover, 16 positive emotions were recognized with higher accuracy from nonverbal vocalizations compared to speech prosody, suggesting a communicative advantage for nonverbal vocalizations. When compared to nonverbal vocalizations, a relative lack of distinctive acoustic cues of positive emotions expressed via prosodic expressions might be leading to poorer recognizability.

### Acoustic Classification Experiments

**Fig. 4:**
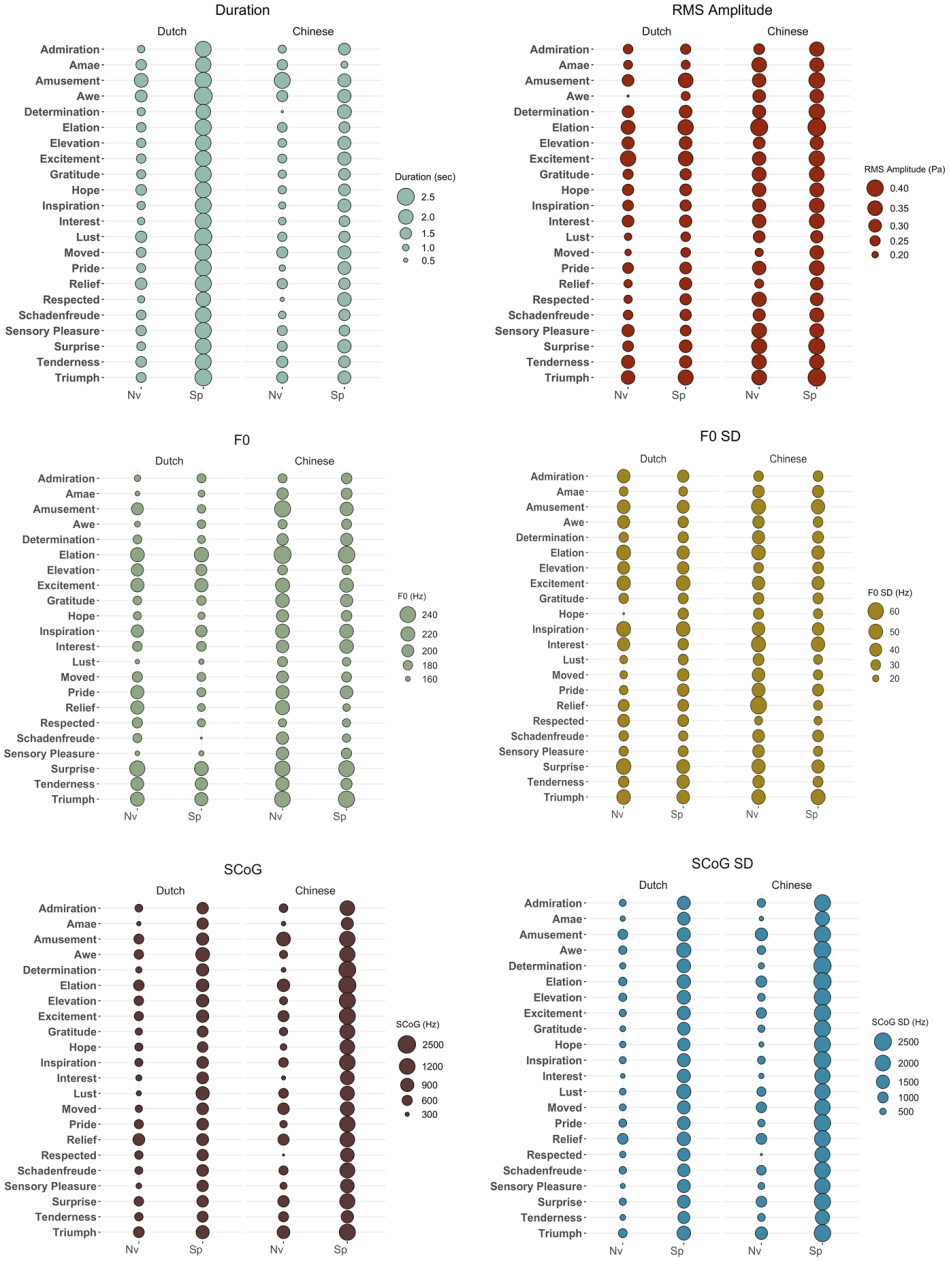
Acoustic characteristics of the vocalizations used in this study. Larger circles signify higher values. Rms = root mean square, SCoG = spectral center of gravity; duration is in seconds, amplitude is in pascal, pitch and spectral measurements are in Hertz. Nv = nonverbal vocalization, Sp = Speech prosody

Machine learning approaches were employed to attempt to automatically classify the nonverbal vocalizations and speech prosody of 22 positive emotions based on their acoustic features. All stimuli collected from the Dutch speakers in Experiment 1 and the Chinese Mandarin speakers in Experiment 2 were used. We first extracted a large number of acoustic features for each audio clip and then performed discriminative classification experiments with machine learning algorithms to try to classify the 22 positive emotions based on the extracted acoustic features. If acoustic classification is higher for nonverbal vocalizations than for speech prosody, this might be one of the contributing mechanisms to better recognition of positive emotions from nonverbal vocalizations in Experiment 1 and 2. The acoustic characteristics of the vocalizations used in this study (duration, Rms amplitude, pitch mean, pitch standard deviation, spectral central of gravity, and spectral standard deviation values, extracted using Praat: Boersma & Weenink, 2011) are presented in Fig. 4.

### Feature Extraction

By utilizing openSMILE software (Eyben et al., 2013), we extracted acoustic features from the extended version of the Geneva Minimalistic Acoustic Parameter Set (eGeMAPs, see Eyben et al., 2016). GeMAPs is a standardized, open source method for measurement of acoustic features for emotional voice analysis. The acoustic features included the frequency, energy/amplitude, spectral balance, and temporal domains. Features of the frequency domain include aspects of fundamental frequency (correlated with the perceived pitch), as well as formant frequencies and bandwidths. Energy/amplitude features refer to the air pressure in the sound wave, and are perceived as loudness. Spectral balance parameters are influenced by laryngeal and supralaryngeal movements and are related to perceived voice quality. Lastly, features from the temporal domain reflect the duration and rate of voiced and unvoiced speech segments. We extracted 88 acoustic features in total from these four domains. For each stimulus, the feature vector was the mean of the whole audio clip.

### Classification Experiments

We conducted acoustic classification experiments with four machine learning algorithms: support vector machine (Linear SVM), linear, radial basis function (RBF SVM), polynomial SVM (Poly SVM), and random forest. These are the most commonly used models for classification (Poria et al., 2017). Scikit-learn, a python-based machine learning library was used for machine learning evaluation (Pedregosa et al., 2011). For all of the machine learning models, we performed tenfold cross-validation and grid search to select the hyperparameters that produced the best results.

We tested classification of 8 positive emotions for each run in order to reflect the findings on human recognition performance in Experiments 1 and 2, in which participants had to select one of 8 emotion options in a forced-choice task. We performed three separate classification runs for all stimuli that had a specific emotion category, henceforth called “emotion category group”. There were 22 emotion category groups corresponding to the 22 emotion categories. First, we used each emotion category group’s actual category plus seven randomly selected emotion categories from the other 21 categories (i.e., excluding the target category). Next, we selected another seven random categories from the remaining 14 categories in addition to the emotion category group’s actual category. Finally, we used the last seven categories and the emotion category group’s actual category. Hence, eight categories were used for each classification run; all 22 categories were included by the end of the third run.

To perform the classification during each run, we split the data into a train-test split using a 60:40% ratio. We optimized our machine learning models on the training set using a hyperparameter grid search. Next, we performed classification on the test set. We then combined the predictions for each of the 22 emotion label groups into one confusion matrix.

### Results

**Fig. 5:**
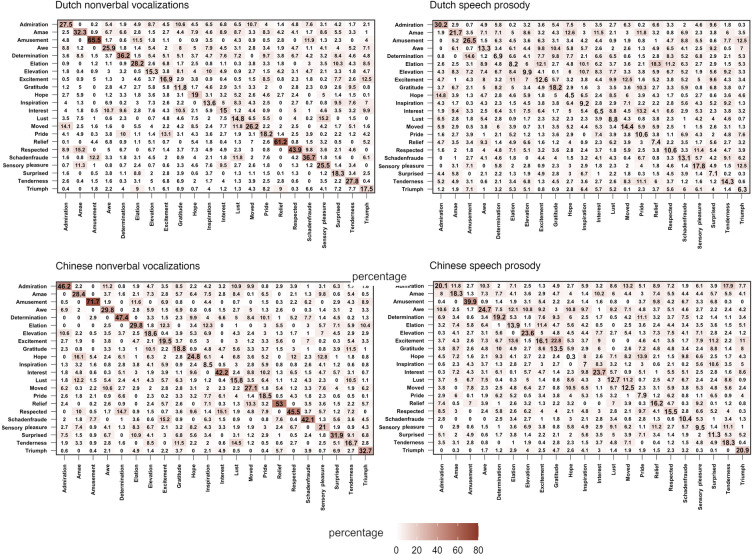
Heatmap of confusion matrices (%) for positive emotions acoustic classification accuracy. The x-axes represent true classification and the y-axes indicate predicted classification

Classification accuracy for each machine learning model is summarized in Table 4; confusion matrices for the most accurate machine learning models for each group are shown in Fig. 5.

**Table 4:** Classification accuracies for each machine learning model

	Dutch		Chinese	
	Nonverbal vocalizations	Speech prosody	Nonverbal vocalizations	Speech prosody
Linear SVM	26.91	**12.79**	25.64	**17.02**
Poly SVM	26.93	11.54	29.41	16.87
RBF SVM	17.06	12.6	18.96	13.2
Random forest	**27.22**	11.59	**31.51**	16.52

Bold mark indicates highest acoustic classification accuracy of each vocalization type expressing 22 positive emotions

For both Dutch and Chinese stimuli, nonverbal vocalizations of all positive emotions except hope and inspiration were classified with above-chance (i.e., 12.5% (1/8) given that there were 8 emotion labels) accuracy. The results revealed that the best classified positive emotions mapped into the emotions well-recognized from nonverbal vocalizations. For speech prosody, only eight positive emotions (admiration, amae, awe, excitement, gratitude, schadenfreude, and tenderness) were classified at above chance levels. Across the machine learning models, nonverbal vocalizations were classified more accurately compared to speech prosody. When vocalization types were compared for each positive emotion, acoustic classification accuracy was higher for nonverbal vocalizations of 18 positive emotions, while none of the emotions were classified with better accuracy from speech prosody. These results illustrate the lower distinctiveness of the acoustic patterns of positive emotions expressed through prosodic expressions as compared to nonverbal vocalizations, providing a likely explanation for the better recognition of positive emotions from nonverbal vocalizations found in Experiment 1 and 2.

### Ancillary Acoustic Analyses

**Fig. 6:**
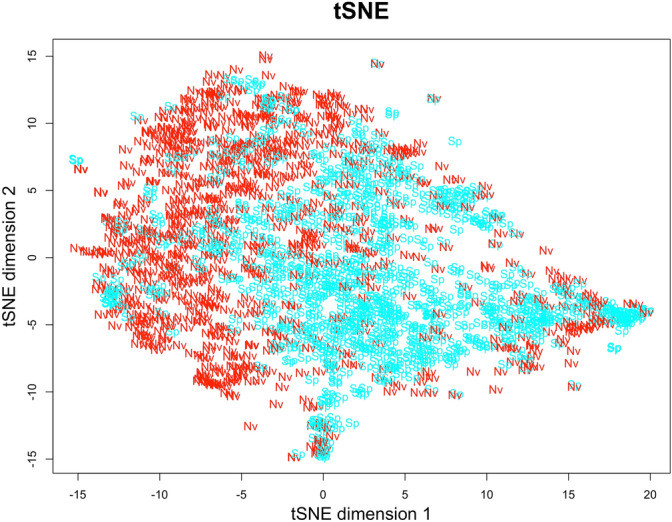
The *t*-distributed stochastic neighbor embedding (*t*-SNE) multidimensional scaling projection of the acoustic structure of positive emotions for each vocalization type. T-SNE estimates local distances between data points without assuming linearity or discreteness. Acoustic structures that are similar are closer in the *t*-SNE space. Nv = nonverbal vocalization, Sp = Speech prosody

In order to better understand acoustic distinctiveness of nonverbal expressions and speech prosody, we first visualised acoustic similarity structure of positive emotions across the two vocalizations types using *t*-distributed stochastic neighbor embedding (*t*-SNE; https://lvdmaaten.github.io/tsne/). In the resulting multidimensional scaling projection, distance between the elements (i.e., acoustic structure of each vocalization) denotes their similarity (see Fig. 6). The similarity space for vocalizations across the two vocalization types derived by t-SNE revealed that nonverbal vocalizations and speech prosody form distinctive clusters.

In order to better understand the acoustic characteristics of nonverbal expressions and speech prosody, we identified the five most important acoustic features based on feature weights. Feature weights represent how much of each of the acoustic features are used by the machine learning model in classifying emotions for nonverbal vocalizations and speech prosody produced by Dutch and Mandarin Chinese speakers. Table 5 lists these parameters together with their definitions, features weights, and standard variations for nonverbal vocalizations and speech prosody, separately. These calculations highlight that feature weights, in general, were higher for nonverbal vocalizations compared to speech prosody. Acoustic features of nonverbal vocalizations were more influential in classification of nonverbal vocalizations compared to speech prosody. Moreover, pitch cues were among the most important cues for speech prosody but not for nonverbal vocalizations, while loudness and spectral-balance cues were among the most important features for both vocalization types. Temporal cues were important in vocal expressions produced by Chinese Mandarin speakers, but not by Dutch individuals. This might reflect differences in linguistic structures across these languages. Specifically, Chinese Mandarin is a syllable-timed language (spacing syllables equally across an utterance) while Dutch is a stress-timed (emphasizing particular stressed syllables at regular intervals) language (e.g., Benton et al., 2007). Most, but not all, of the acoustic features have more variation in nonverbal vocalizations compared to speech prosody. This could be due to linguistic constraints in the production of speech prosody. The production of nonverbal vocalizations—unlike speech—does not require precise movements of articulators, because they are not constrained by linguistic codes (Scott et al., 2010, see General Discussion for a discussion).

**Table 5:** Five acoustic features with highest feature weights for each condition

Feature type	Description	Feature weight (*SD*_Nv_,*SD*_Sp_)			
		Dutch Nv	Dutch Sp	Chinese Nv	Chinese Sp
*Pitch cues*					
F0PercRage0-2	Range of the 20th to 80th percentile of F0 on a semitone frequency scale				Feature 2: 0.0154 (8.12, 5.75)
F0SlopeFallSD	Standard deviation of the falling slopes of F0		Feature 1: 0.0159 (141.02, 209.60)		
F3BandwidthSD	Bandwidth standard deviation of third formant				Feature 1: 0.0155 (0.18, 0.1)
*Loudness cues*					
LoudnessPerc50	50th percentile of loudness	Feature 5: 0.0167 (1.22, 1.16)	Feature 5: 0.0143 (1.22, 1.16)		
LoudnessSlopeRise	Mean of the rising slopes of loudness	Feature 3: 0.0178 (13.02, 11.21)		Feature 2: 0.0195 (8.12, 5.75)	
LoudnessSlopeFall	Mean of the falling slopes of loudness	Feature 1: 0.0196 (10.80, 11.21)		Feature 5: 0.0158 (12.92, 13)	Feature 5: 0.0145 (12.92, 13)
LoudnessSlopeFallSD	Standard deviation of the falling slopes of loudness		Feature 2: 0.0151 (7.75, 6.73)		
*Spectral-balance cues*					
Mfcc1V	Mean of mel-frequency cepstral coefficient 1 of voiced regions			Feature 4: 0.0164 (8.32, 5.19)	
Mfcc2V	Mean of mel-frequency cepstral coefficient 2 of voiced regions		Feature 3: 0.0145 (8.46, 4.57)		
Mfcc3	Mean of mel frequency cepstral coefficient 3	Feature 4: 0.0167 (9.65, 4.17)			
Mfcc3V	Mean of mel frequency cepstral coefficient 3 of voiced regions	Feature 2: 0.0179 (11.23, 5.75)	Feature 4: 0.0143 (11.23, 5.75)		
SpectralFluxV	Mean of the difference of the spectra of two consecutive frames of voiced regions			Feature 3: 0.0165 (2.52, 2.05)	
*Temporal cues*					
LoudnessPeaks	The number of loudness peaks per second			Feature 1: 0.0226 (1.10, 0.97)	Feature 3: 0.0147 (1.10, 0.97)
UnvoicedSegSD	Standard deviation of unvoiced regions (approximating pauses)				Feature 4: 0.0147 (0.18, 0.06)

For more information on the definitions of acoustic features see Eyben et al. (2016) and Pajupuu, Altrov, and Pajupuu, 2019

We further performed cross-classification analyses in order to test whether producers’ emotion encoding strategies overlap between nonverbal vocalizations and speech prosody, and whether the encoding strategies are shared across Dutch and Chinese Mandarin speaking participants. For the cross-classification analyses we thus conducted two types of analyses: (1) trained models on nonverbal vocalizations and tested on speech prosody, and vice versa; (2) trained models on Dutch speaking participants vocalizations and tested on vocalizations produced by Chinese Mandarin speaking participants, and vice versa. The accuracy of all models are shown in Table 6.

**Table 6:** Classification accuracies for cross-vocalizations type and cross-cultural evaluations

	Dutch				Chinese			
	Cross-vocalization type evaluation		Cross-cultural evaluation		Cross-vocalization type evaluation		Cross-cultural evaluation	
	Training on Nv, testing on Sp	Training on Sp, testing on Nv	Training on Dutch, testing on Chinese		Training on Nv, testing on Sp	Training on Sp, testing on Nv	Training on Chinese, testing on Dutch	
			Nv	Sp			Nv	Sp
Linear SVM	12.63	13.45	27.47	14.93	16.69	21.12	22.7	12.21
Poly SVM	12.66	12.31	28.93	13.94	17.28	19.1	27.71	14.59
RBF SVM	13.92	11.25	24.58	**15.12**	15.4	21.87	17.26	12.84
Random forest	**14.01**	**13.62**	**31.85**	14.59	**17.48**	**24.08**	**30.4**	**15.48**

Bold mark indicates best performance. Classification accuracy is higher than the chance level (i.e., 12.5%). Nv = nonverbal vocalizations, Sp = speech prosody

The results show that classification models in each of the cross-classification types performed statistically better than chance, indicating shared encoding strategies used in the production of emotional vocalizations. In cross-vocalization type evaluations, performance was nearly equivalent for training and test in both directions for the Dutch vocal expressions. However, for the vocalizations produced by Chinese Mandarin speakers, the accuracies were slightly higher for training on speech prosody and testing on nonverbal vocalizations as compared to the reverse. In cross-cultural evaluations, training on the Dutch vocalizations and testing on Chinese vocalizations performed similarly as training on the Chinese vocalizations and testing on Dutch vocalizations. Cross-cultural classification performance was better for nonverbal vocalizations compared to speech prosody, suggesting more robust differentiation of positive emotions based on acoustic configurations across cultures when expressed via nonverbal vocalizations. Cross-classification evaluations demonstrate that encoding strategies used in production of emotional vocalizations shared across vocalization types as well as the speakers from the two cultures.

## General Discussion

The current study demonstrates that nonverbal vocalizations expressing a wide range of positive emotions hold a communicative advantage over prosodic expressions. We examined recognition of 22 positive emotions from nonverbal vocalizations and speech prosody in two distant cultural contexts. The results showed differential accuracy depending on the vocalization type through which positive emotions were expressed. In particular, listeners recognized emotions better from nonverbal vocalizations compared to speech prosody. This pattern was found for many but not all positive emotions, and so the superiority of nonverbal vocalizations is influenced by the specific positive emotion expressed in the voice.

### Recognition of Positive Emotions from Nonverbal Vocalizations Versus Speech Prosody

The current study adds to the scant knowledge available on differences in emotion recognition between types of vocal expressions. Our results show that most positive emotions are better recognized from brief nonverbal vocalizations than from speech prosody. This demonstrates that nonverbal vocalizations can communicate positive emotions more successfully than speech prosody, even though nonverbal vocalizations are considerably shorter in duration. Brief nonverbal vocalizations are more densely packed with emotional information compared to speech prosody. Previous research has reported better recognition from nonverbal vocalizations as compared to speech prosody for several negative emotions and for happiness/joy (e.g., Hawk et al., 2009). Our study, for the first time, provides comparisons of recognition for different vocalization types for a wide range of positive emotions. The results imply that nonverbal vocalizations may be richer and more nuanced than previously thought, given the wide range of positive emotions that could be clearly conveyed via such cues.

The results of cross-classification analysis with machine learning models show shared encoding strategies in production of emotional vocalizations across vocalization types and cultures. Dutch and Chinese Mandarin speakers employed shared mechanisms in production of both nonverbal vocalizations and speech prosody. Furthermore, cross-cultural classification evaluations show that differentiation of positive emotions based on acoustic features was more robust across cultures when expressed as nonverbal vocalizations compared to speech prosody. Indeed, the results of acoustic analysis with machine learning models demonstrate that acoustic configurations of discrete positive emotions were highly differentiated from those of other emotions when expressed through nonverbal vocalizations but less so for speech prosody. Discriminability of acoustic patterns in nonverbal expressions of positive emotions paralleled human listeners’ patterns of recognition accuracy. One possibility is that the superiority of nonverbal vocalization in recognition of positive emotions might be due to more distinctive acoustic patterns.

### Communicating Positive Emotions via Nonverbal Vocalizations

Both Dutch and Chinese nonverbal vocalizations were highly effective means of communicating 11 different positive emotions. These results are in line with previous research showing that amusement, interest, lust, relief, and surprise are well-recognized from nonverbal vocalizations (e.g., Cordaro et al., 2016; Cowen et al., 2019; Laukka et al., 2013). In addition to these emotions, the current investigation showed that nonverbal vocalizations can reliably communicate admiration, determination, excitement, inspiration, schadenfreude, and sensory pleasure. The recognition scores for nonverbal vocalization of schadenfreude and sensory pleasure are particularly notable, given the low recognition rates of these emotions from speech prosody. Nonverbal vocalizations of these positive emotions appear to map onto distinct, recognizable vocal signatures. Within a functional framework, different positive emotions serve adaptive functions relating to different types of opportunities, like affiliation and cooperation (e.g., Fredrickson, 1998; Griskevicius et al., 2010; Keltner et al., 2006; Shiota et al., 2004, 2014). For instance, schadenfreude has been proposed to serve a social affiliation function by strengthening ingroup bonds (Yam, 2017), and sensory pleasure motivates an individual to pursue reward necessary for fitness (Berridge & Kringelbach, 2015). Based on the highest recognition accuracies of positive emotions in both cultures (see Fig. 1), more clearly recognized nonverbal vocalizations are vocalizations of amusement, relief, schadenfreude, sensory pleasure, and surprise.

### Communicating Positive Emotions via Speech Prosody

For speech prosody, participants were able to recognize only amusement, determination, relief, and triumph with above chance accuracy in both cultural contexts. These emotions might be associated with prosodic configurations in running speech that are highly differentiable from other positive emotions. For instance, when expressing amusement via speech prosody, we might produce salient prosodic expressions which might signal cooperative intent to others. The accuracy rate of successfully recognized positive emotions, as well as the overall recognition rate, were lower for speech prosody compared to nonverbal vocalizations. Prosodic expressions require more complex coordination of articulators with greater volitional control due to linguistic structures in speech. In contrast, nonverbal vocalizations are produced with less volitional control while articulators are mostly in their resting positions. The lack of constraints on nonverbal expressions allows more flexibility in the expression of emotions, avoiding the linguistic constraints that exist in prosodic expressions. Despite the lower recognition accuracy, specific positive emotions could be recognized from speech prosody in both languages. Previous literature has shown that emotions like anger, sadness, and fear can be recognized from speech prosody across languages, and certain acoustic features such as speaker fundamental frequency have great importance in signaling these emotions (e.g., Paulmann & Uskul, 2014; Pell et al., 2009). Our results suggest that prosodic expressions of amusement, determination, and relief future are well-recognized based on their high recognition accuracies of positive emotions in both cultures (see Fig. 1).

Previous research on emotional speech prosody have found higher levels of recognition accuracy compared to those in the present study (e.g., Hawk et al., 2009; Pell et al., 2009; Scherer et al., 1991). There are several potential reasons for this difference. One possibility is that discrete positive emotions might be recognized with lower accuracy levels from speech prosody than the primarily negative emotions studied in previous research. Previous studies mostly included a general positive emotion category (i.e., happiness/joy) and discrete negative emotions like anger, fear, and sadness. It is likely that comparing these emotions with each other was easier for participants as compared to our study. In addition, lower numbers of emotion categories were included in most previous studies. Given that participants compared eight positive emotions in our study, the difficulty of the emotion recognition task could explain the lower recognition accuracy in our study. Methodological differences may also have contributed to these differences in recognition accuracy. The present study included all stimuli, whereas previous studies have pre-selected stimuli based on listeners’ judgments. For instance, in the study of Pell et al. (2009), only stimuli that were recognized at minimally three times chance performance were included in the analysis of overall recognition levels. In the study of Hawk et al. (2009), two raters evaluated all stimuli and selected the ones for which there was good correspondence with the target emotion. Applying such pre-selection criteria is certain to inflate emotion recognition accuracy.

### Cultural Differences in Recognition of Positive Emotions

The recognizability of some positive emotions from nonverbal vocalizations and speech prosody differed between the two cultural contexts. For instance, amae was recognized from Chinese but not Dutch nonverbal vocalizations. Amae is an emotion originating in an East Asian context (Doi, 2005), loosely translated as attachment love in English. One possibility is that amae may have normative vocal expressions in Chinese culture, but not in the Dutch cultural context. For speech prosody, we found that being respected was recognized only by Dutch listeners, while awe was recognized only in the Chinese culture. These findings suggest that prototypical prosodic expressions can exist in one culture without necessarily occurring in other cultural contexts.

The present study only assessed within-culture recognition, that is, producer and perceiver came from the same culture. Repeating the experiment in two cultural contexts sought to test whether the findings would be replicable. However, this approach precludes the investigation of cross-cultural recognition of positive emotions. Findings from previous studies point to impairments in cross-cultural recognition of happiness from vocal expressions (see Laukka & Elfenbein, 2020 for a meta-analysis). These challenges are more pronounced than in the cross-cultural communication of vocal expressions of negative emotions, suggesting that positive vocalizations might be particularly susceptible to cultural differences. For instance, Pell and colleagues (2009) showed that, across four languages, acoustic features extracted from prosodic expressions of happiness were more variable than those of negative emotions like disgust and fear. Sauter et al. (2010) suggested that vocal signals of negative emotions might be less influenced by cultural learning compared to positive emotions. Negative signals may be more related to biological reactions to immediate dangers, while communication of positive emotions might facilitate social capacities that promote adaptation (e.g., Fredrickson, 1998; Nesse, 1990). Further work is needed to test the extent to which the challenges with cross-cultural communication of vocal signals is true of the wide range of positive emotions examined in the present study. Some evidence has found that laughter is well recognized as communicating amusement across cultural groups (Sauter et al., 2010), suggesting that there is likely considerable variability across positive emotions and expression types. Research that includes the production and perception of a wide range of positive emotions will be needed to establish this empirically.

### Limitations and Future Research Suggestions

Our study has several limitations that merit consideration. One point is that we used a forced-choice design to assess recognition. Forced-choice tasks provide a convenient way to collect and analyze categorical data. However, they might potentially inflate perceiver accuracy because participants can use guessing strategies that are informed by the available response alternatives (e.g., Russell, 1994). Most studies testing recognition of emotions from nonverbal expressions used forced-choice methodology and included a relatively small number of response alternatives (e.g., four: Cordaro et al., 2020; Scherer et al., 2001). In such tasks, comparing small number of emotions to make a judgment might artificially inflate recognition rates by enabling informed guessing strategies. Increasing the number of alternatives in a forced-choice task might reduce the guessing rate, but there is also a point at which the number of alternatives becomes too large for perceivers (Vancleef et al., 2018). In the present study, we considered that it would be too cumbersome for participants to choose between 22 response options. We, therefore, opted to let participants select from eight positive emotion categories, with response options different across trials. Generalized linear mixed models with responses to such a task allowed us to assess recognition of 22 positive emotions, while keeping the number of response options at a manageable level. However, we cannot rule out that participants may have been able to make use of elimination strategies to help guide their responses on some trials.

Another limitation is that we used posed expressions of positive emotions. The vocalizations were produced by untrained individuals who were asked to produce vocal expressions of specific emotions on demand. Posed vocalizations provide better sound quality since they can be recorded with high-quality equipment in the lab, while it is typically challenging to record good-quality audio from spontaneous vocal expressions in real-world contexts. In addition to better sound quality, posed vocalizations also afford certainty about the intended emotion being expressed. We provided definitions and situational examples in order to ensure that the expressions were targeting the intended emotions. However, the producers did not experience those emotions when producing the vocalizations. In contrast, spontaneous vocalizations occurring in real-world settings are more natural and thus have the advantage of reflecting genuinely felt emotions (Williams & Stevens, 1981). Previous research points to some differences in acoustic properties of spontaneous and posed emotional vocalizations (e.g., Anikin & Lima, 2018). For instance, spontaneous laughter typically has higher pitch and shorter burst duration compared to volitional laughter (e.g., Bryant & Aktipis, 2014). For emotional speech, most acoustic features show similar patterns for spontaneous and posed speech, while some subtle acoustic differences have been found in measures of frequency and temporal features (e.g., Juslin et al., 2017). It is thus possible that some acoustic characteristics of the vocalizations used in our study differ from those of spontaneous vocalizations of the same emotions, and recognizability of positive emotions might even be stronger for spontaneous vocalizations (Anikin & Lima, 2018, but see Sauter & Fischer, 2018). In order to investigate the acoustic profiles and recognition accuracy of positive emotions in vocal expressions that are higher in ecological validity, future research should aim to collect high-quality recordings of spontaneous vocalizations of different positive emotions in real life (e.g., Anderson et al., 2018).

Examining (filtered versions of) spontaneously produced emotional speech would allow researchers to avoid the potential artifact of imposing standardized utterances. Encoding and decoding of emotions in speech prosody might be influenced by the use of standardized semantic content. The use of standard utterances such as names and pseudo-speech is common in the study of emotional prosody (see Juslin & Laukka, 2003 for a review). In our study, producing a number, “six hundred forty-seven,” in an emotionally inflected way might have felt unnatural and thus been difficult for speakers. Similarly, recognition of emotions from such stimuli might have been an unfamiliar, and thus challenging task for the listeners. The use of a standardized utterance may have hampered the production and perception of emotional speech, which could have contributed to the poorer recognition of emotions from speech prosody compared to nonverbal vocalizations (although producing nonverbal vocalizations on demand may have felt unnatural for participants too). Future research could examine the distinctiveness of acoustic features of emotions in spontaneous speech. Additionally, the emotion recognition ability of listeners who are from a close culture but do not understand the language spoken could be employed to address the contribution of difficulties in producing semantically constrained speech.

In producing nonverbal vocalizations of positive emotions, encoders sometimes used emblems like “wow” that are culturally shaped, conventionalized vocalizations (Scherer, 1994; see https://emotionwaves.github.io/dutch22/ to listen some examples). Since emblematic vocalizations are culturally bound and convey a symbolic meaning, it may be plausible to expect that emblematic vocalizations are more accurately recognized than raw vocalizations (when producer and perceiver are from the same culture). Previous research, however, has shown higher decoding accuracy for both raw and emblematic vocalizations compared to speech prosody (e.g., compare Schröder, 2003 with Banse & Scherer, 1996; Hawk et al., 2009). Moreover, the distinction between raw and emblematic vocalizations is far from clear-cut. While nonverbal vocalizations like laughs and screams are considered relatively raw expressions that naturally occur, emblematic expressions are more likely to be produced when the communication is intentional (Buck, 1984). However, affective vocal signals conveyed in everyday life are suggested to fall somewhere on a continuum between raw and emblematic, rather than being one or the other. The distinction between raw and emblematic expressions is likely even less evident when considering real-world vocalizations of emotions because “all sorts of mixtures” occur (e.g., Banse & Scherer, 1996; Scherer, 1994; Schröder, 2003). The setup of our study further blurs the line between these expressions, because not only the emblematic expressions, but all of the nonverbal vocalizations were produced intentionally. In order to draw firmer conclusions about what constitutes emblematic vocalizations of positive emotions and the proportional use of emblems in vocal expression, future research should gather more emblematic vocalizations, perhaps across different cultures.

The acoustic classification performed slightly worse than the human listeners overall. One reason for poorer classification accuracy might be the small dataset in our study. Further approaches can be explored in the future to improve the machine learning results, such as deep neural networks in which the network is trained on a different but related task that has large number of examples. Another reason contributing to the slightly worse performance of the acoustic classification might be the features used in the training of the classifier. Dataset used in our study mostly involved the arithmetic mean of the extracted features. Classifiers trained on datasets including temporal information that characterizes vocal expressions might provide better performance.

### Conclusions

In conclusion, we provide evidence for systematic differences between different kinds of vocal expressions of positive emotions. Overall, the results of this study demonstrate the superiority of nonverbal vocalizations over speech prosody for recognition of many positive emotions. We demonstrate that positive emotions are also expressed with more distinctive acoustic patterns in nonverbal vocalizations as compared to speech prosody. Finally, our results show that human listeners can accurately perceive a wide range of positive emotions from nonverbal vocalizations but only a few from speech prosody.

## Supplementary Information

Below is the link to the electronic supplementary material.

Supplementary file 1 (DOCX 27 kb)

## Data Availability

Data and code are available from https://osf.io/djgq9/?view_only=6ba8056f2f564d4b9d9d21b9c4014de4.

## References

[CR1] Ameka F (1992). Interjections: The universal yet neglected part of speech. Journal of Pragmatics.

[CR2] Anderson CL, Monroy M, Keltner D (2018). Emotion in the wilds of nature: The coherence and contagion of fear during threatening group-based outdoors experiences. Emotion.

[CR3] Anikin A, Lima CF (2018). Perceptual and acoustic differences between authentic and acted nonverbal emotional vocalizations. The Quarterly Journal of Experimental Psychology.

[CR4] Banse R, Scherer KR (1996). Acoustic profiles in vocal emotion expression. Journal of Personality and Social Psychology.

[CR5] Bates D, Mächler M, Bolker B, Walker S (2015). Fitting linear mixed-effects models using lme4. Journal of Statistical Software.

[CR6] Behrens KY (2004). A multifaceted view of the concept of amae: Reconsidering the indigenous Japanese concept of relatedness. Human Development.

[CR7] Benton, M., Dockendorf, L., Jin, W., Liu, Y., & Edmondson, J. A. (2007). The continuum of speech rhythm: Computational testing of speech rhythm of large corpora from natural Chinese and English speech. *The 16th ICPhS* (pp. 1269–1272).

[CR8] Berridge KC, Kringelbach ML (2015). Pleasure systems in the brain. Neuron.

[CR9] Boersma, P., & Weenink, D. (2011). *Praat: Doing phonetics by computer.* Retrieved from http://www.praat.org/.

[CR10] Bryant GA, Aktipis CA (2014). The animal nature of spontaneous human laughter. Evolution and Human Behavior.

[CR11] Buck R (1984). The communication of emotion.

[CR12] Castiajo P, Pinheiro AP (2019). Decoding emotions from nonverbal vocalizations: How much voice signal is enough?. Motivation and Emotion.

[CR13] Cordaro DT, Keltner D, Tshering S, Wangchuk D, Flynn LM (2016). The voice conveys emotion in ten globalized cultures and one remote village in Bhutan. Emotion.

[CR14] Cordaro DT, Sun R, Kamble S, Hodder N, Monroy M, Cowen A, Bai Y, Keltner D (2020). The recognition of 18 facial-bodily expressions across nine cultures. Emotion.

[CR15] Cowen AS, Elfenbein HA, Laukka P, Keltner D (2019). Mapping 24 emotions conveyed by brief human vocalization. American Psychologist.

[CR16] Doi T (2005). Understanding amae: The Japanese concept of need-love.

[CR17] Ekman P (1992). An argument for basic emotions. Cognition and Emotion.

[CR18] Eyben F, Scherer KR, Schuller BW, Sundberg J, André E, Busso C, Devillers LY, Epps J, Laukka P, Narayanan SS, Truong KP (2016). The Geneva Minimalistic acoustic parameter set (GeMAPS) for voice research and affective computing. IEEE Transactions on Affective Computing.

[CR19] Eyben, F., Weninger, F., Gross, F., & Schuller, B. (2013). Recent developments in openSMILE, the Munich open-source multimedia feature extractor. In A. Jaimes, N. Sebe, N. Boujemaa, D. Gatica-Perez, D. A. Shamma, M. Worring, & R. Zimmermann (Eds.), *Proceedings of the 21st association for computing machinery international conference on multimedia* (pp. 835–838). New York, NY: Association for Computing Machinery. 10.1145/2502081.2502224

[CR20] Fredrickson BL (1998). What good are positive emotions?. Review of General Psychology.

[CR21] Gibbon, D. (2017). *Prosody: Rhythms and melodies of speech*. Retrieved from https://arxiv.org/pdf/1704.02565.pdf

[CR22] Griskevicius V, Shiota MN, Neufeld SL (2010). Influence of different positive emotions on persuasion processing: A functional evolutionary approach. Emotion.

[CR23] Hawk ST, Van Kleef GA, Fischer AH, Van Der Schalk J (2009). "Worth a thousand words": Absolute and relative decoding of nonlinguistic affect vocalizations. Emotion.

[CR24] Jessen S, Kotz SA (2011). The temporal dynamics of processing emotions from vocal, facial, and bodily expressions. NeuroImage.

[CR25] ﻿Juslin, P. N., & Laukka, P.  (2001). Impact of intended emotion intensity on cue utilization and decoding accuracy in vocal expression of emotion. Emotion.

[CR26] Juslin PN, Laukka P (2003). Communication of emotions in vocal expression and music performance: Different channels, same code?. Psychological Bulletin.

[CR27] Juslin PN, Laukka P, Bänziger T (2017). The mirror to our soul? Comparisons of spontaneous and posed vocal expression of emotion. Journal of Nonverbal Behavior.

[CR28] Kamiloğlu RG, Fischer AH, Sauter DA (2020). Good vibrations: A review of vocal expressions of positive emotions. Psychonomic Bulletin & Review.

[CR29] Keltner D, Haidt J, Shiota MN, Schaller M, Simpson JA, Kenrick DT (2006). Social functionalism and the evolution of emotions. Evolution and social psychology.

[CR30] Kreiman J, Sidtis D (2011). Foundations of voice studies: An interdisciplinary approach to voice production and perception.

[CR31] Laukka P, Elfenbein HA (2020). Cross-cultural emotion recognition and in-group advantage in vocal expression: A meta-analysis. Emotion Review.

[CR32] Laukka P, Elfenbein HA, Söder N, Nordström H, Althoff J, Iraki FKE, Rockstuhl T, Thingujam NS (2013). Cross-cultural decoding of positive and negative non-linguistic emotion vocalizations. Frontiers in Psychology.

[CR33] Lausen A, Hammerschmidt K (2020). Emotion recognition and confidence ratings predicted by vocal stimulus type and prosodic parameters. Humanities and Social Sciences Communications.

[CR34] Lima CF, Castro SL, Scott SK (2013). When voices get emotional: A corpus of nonverbal vocalizations for research on emotion processing. Behavior Research Methods.

[CR35] Nesse RM (1990). Evolutionary explanations of emotions. Human Nature.

[CR36] Pajupuu, H., Altrov, R., & Pajupuu, J. (2019). Towards a vividness in synthesized speech for audiobooks. Eesti ja soome-ugri keeleteaduse ajakiri. *Journal of Estonian and Finno-Ugric Linguistics, 10*, 167–190. 10.12697/jeful.2019.10.1.09

[CR37] Panksepp J, Burgdorf J (2003). “Laughing” rats and the evolutionary antecedents of human joy?. Physiology & Behavior.

[CR38] Paulmann S, Uskul AK (2014). Cross-cultural emotional prosody recognition: Evidence from Chinese and British listeners. Cognition and Emotion.

[CR39] Pedregosa F, Varoquaux G, Gramfort A, Michel V, Thirion B, Grisel O, Blondel M, Prettenhofer P, Weiss R, Dubourg V, Vanderplas J (2011). Scikit-learn: Machine learning in Python. The Journal of Machine Learning Research.

[CR40] Pell MD, Paulmann S, Dara C, Alasseri A, Kotz SA (2009). Factors in the recognition of vocally expressed emotions: A comparison of four languages. Journal of Phonetics.

[CR41] Pell MD, Rothermich K, Liu P, Paulmann S, Sethi S, Rigoulot S (2015). Preferential decoding of emotion from human non-linguistic vocalizations versus speech prosody. Biological Psychology.

[CR42] Poria S, Cambria E, Bajpai R, Hussain A (2017). A review of affective computing: From unimodal analysis to multimodal fusion. Information Fusion.

[CR43] Russell JA (1994). Is there universal recognition of emotion from facial expression? A review of the cross-cultural studies. Psychological Bulletin.

[CR44] Sauter, D. (2007). *An investigation into vocal expressions of emotions: The roles of valence, culture, and acoustic factors* (Doctoral dissertation, University of London).

[CR45] Sauter DA, Eisner F, Ekman P, Scott SK (2010). Cross-cultural recognition of basic emotions through nonverbal emotional vocalizations. Proceedings of the National Academy of Sciences of the United States of America.

[CR46] Sauter DA, Fischer AH (2018). Can perceivers recognize emotions from spontaneous expressions?. Cognition and Emotion.

[CR47] Schaerlaeken S, Grandjean D (2018). Unfolding and dynamics of affect bursts decoding in humans. PLoS ONE.

[CR48] Scherer KR (1986). Vocal affect expression: A review and a model for future research. Psychological Bulletin.

[CR49] Scherer KR, van Goozen SHM, Van de Poll NE, Sergeant JA (1994). Affect bursts. Emotions: Essays on emotion theory.

[CR50] Scherer KR, Banse R, Wallbott HG (2001). Emotion inferences from vocal expression correlate across languages and cultures. Journal of Cross-Cultural Psychology.

[CR51] Scherer KR, Banse R, Wallbott HG, Goldbeck T (1991). Vocal cues in emotion encoding and decoding. Motivation and Emotion.

[CR52] Schröder M (2003). Experimental study of vocal affect bursts. Speech Communication.

[CR53] Scott, S. K., Sauter, D., & McGettigan, C. (2010). Brain mechanisms for processing perceived emotional vocalizations in humans. In *Handbook of behavioral neuroscience* (Vol. 19, pp. 187–197). Elsevier.

[CR54] Shiota MN, Campos B, Keltner D, Hertenstein M, Phillipot P, Feldman R (2004). Positive emotion and the regulation of interpersonal relationships. Emotion regulation.

[CR55] Shiota MN, Neufeld SL, Danvers AF, Osborne EA, Sng O, Yee CI (2014). Positive emotion differentiation: A functional approach. Social and Personality Psychology Compass.

[CR56] Soltysik S, Jelen P (2005). In rats, sighs correlate with relief. Physiology & Behavior.

[CR57] Trouvain J (2014). Laughing, breathing, clicking—The prosody of nonverbal vocalizations. Speech Prosody.

[CR58] Vancleef K, Read JC, Herbert W, Goodship N, Woodhouse M, Serrano-Pedraza I (2018). Two choices good, four choices better: For measuring stereoacuity in children, a four-alternative forced-choice paradigm is more efficient than two. PLoS ONE.

[CR59] Williams CE, Stevens KN, Darby JK (1981). Vocal correlates of emotional states. Speech evaluation in psychiatry.

[CR60] Yam, P. C. (2017). *The social functions of intergroup schadenfreude* (Doctoral dissertation, University of Oxford).

