# Supplementary material for: Superior Communication of Positive Emotions Through Nonverbal Vocalisations Compared to Speech Prosody
Source: J Nonverbal Behav. 2021 Jul 24;45(4):419–54. doi: 10.1007/s10919-021-00375-1 (PMC8553689; doi:10.1007/s10919-021-00375-1)
Supplement: Supplementary file 1 — (DOCX 27 kb) [file 10919_2021_375_MOESM1_ESM.docx]

**Supplementary Materials**

**Script 1S**

library(lme4)

library(magrittr)

library(dplyr)

library(tidyr)

library (tibble)

logit = function(p) log(p / (1-p))

ilogit = function(y) 1/(1+exp(-y))

## The item difficulty is computed as:

## item difficulty = alpha + beta * (1:22) + rnorm()

## change alpha and beta to specify anticipated effect sizes on the log-scale

alpha = 0.05 # set to > 10 to simulate pure random guessing; small values for effects

beta = 0.1 # set to 0 for no difference between emotions

stims = expand.grid(expr = factor(1:2), emo=factor(1:22)) %>%

mutate(difficulty = rnorm(expr)+alpha+beta*as.integer(emo)) %>%

rownames_to_column('vid')

## Data are simulated for n judges; increase n to increase power

n = 200

nreps <- 2 + rep(0:1, c(44-12,12)) # number of repetitions within expr*emo cell

simdata <-

expand.grid(sid=factor(1:n), expr=factor(1:2), emo=factor(1:22), rep=1:3) %>%

group_by(sid) %>%

arrange(expr, emo) %>%

mutate(m = rep(sample(nreps), each=3), ability = rnorm(n)[as.integer(sid)]) %>%

filter(rep <= m) %>%

ungroup() %>%

arrange(sid, expr, emo) %>%

select(-rep,-m) %>%

left_join(stims) %>%

ungroup() %>%

mutate(P=1/8+(7/8)*ilogit(ability-difficulty), guessC=logit(1/8), resp=P>runif(P))

glmer(resp ~ offset(guessC) + emo + (1|vid) + (1|sid), data=simdata, family = binomial) %>%

glmer(resp ~ offset(guessC) + expr + (1|vid) + (1|sid), data=simdata, family = binomial) %>%

summary()

**Table 1S.** Fixed effects in GLMM models comparing recognition accuracy to chance level per emotion

| **Fixed Effects** | **Dutch nonverbal vocalizations** | | | | **Dutch speech prosody** | | | | **Chinese nonverbal vocalizations** | | | | **Chinese speech prosody** | | | | |
| --- | --- | --- | --- | --- | --- | --- | --- | --- | --- | --- | --- | --- | --- | --- | --- | --- | --- |
|  | **Est.** | ***SE*** | ***Z*** | ***p* (>\|*z*\|)** | **Est.** | ***SE*** | ***Z*** | ***p* (>\|*z*\|)** | **Est.** | ***SE*** | ***Z*** | ***p* (>\|*z*\|)** | **Est.** | ***SE*** | ***Z*** | ***p* (>\|*z*\|)** |  |
| Admiration | 2.099 | 0.331 | 6.336 | **<0.001** | 0.753 | 0.240 | 3.135 | **0.002** | 0.821 | 0.275 | 2.982 | **0.003** | -0.479 | 0.265 | -1.807 | 0.071 |  |
| Amae | -0.945 | 0.489 | -1.934 | 0.053 | -0.084 | 0.353 | -0.239 | 0.811 | 2.319 | 0.410 | 5.651 | **<0.001** | 0.533 | 0.392 | 1.361 | 0.174 |  |
| Amusement | 2.484 | 0.378 | 6.576 | **<0.001** | 1.401 | 0.453 | 3.092 | **0.002** | 3.587 | 0.324 | 11.08 | **<0.001** | 1.453 | 0.284 | 5.125 | **<0.001** |  |
| Awe | 1.170 | 0.330 | 3.541 | **<0.001** | 0.397 | 0.241 | 1.642 | 0.101 | 0.381 | 0.245 | 1.553 | 0.12 | 0.465 | 0.205 | 2.271 | **0.023** |  |
| Determination | 1.451 | 0.166 | 8.726 | **<0.001** | 1.725 | 0.198 | 8.721 | **<0.001** | 2.123 | 0.322 | 6.586 | **<0.001** | 1.165 | 0.244 | 4.775 | **<0.001** |  |
| Elation | 0.339 | 0.408 | 0.83 | 0.407 | 0.567 | 0.309 | 1.838 | 0.066 | 0.762 | 0.259 | 2.937 | **0.003** | 0.297 | 0.297 | 0.999 | 0.318 |  |
| Elevation | -0.234 | 0.187 | -1.254 | 0.21 | -0.367 | 0.253 | -1.449 | 0.147 | 0.692 | 0.248 | 2.792 | **0.005** | 0.009 | 0.298 | 0.031 | 0.975 |  |
| Excitement | 1.308 | 0.289 | 4.522 | **<0.001** | 0.454 | 0.001 | 339.1 | **<0.001** | 0.604 | 0.269 | 2.246 | **0.025** | 0.191 | 0.258 | 0.74 | 0.459 |  |
| Gratitude | 0.176 | 0.259 | 0.68 | 0.497 | -0.485 | 0.394 | -1.231 | 0.218 | -0.412 | 0.386 | -1.069 | 0.285 | -1.226 | 0.567 | -2.164 | 0.03 |  |
| Hope | -0.855 | 0.430 | -1.988 | 0.047 | -0.556 | 0.423 | -1.313 | 0.189 | -0.725 | 0.515 | -1.407 | 0.159 | 0.136 | 0.201 | 0.675 | 0.5 |  |
| Inspiration | 0.628 | 0.264 | 2.384 | **0.017** | -0.505 | 0.418 | -1.208 | 0.227 | 0.751 | 0.238 | 3.159 | **0.002** | -0.262 | 0.264 | -0.99 | 0.322 |  |
| Interest | 1.233 | 0.244 | 5.061 | **<0.001** | 0.378 | 0.234 | 1.615 | 0.106 | 1.931 | 0.264 | 7.307 | **<0.001** | 0.662 | 0.200 | 3.311 | **<0.001** |  |
| Lust | 1.791 | 0.376 | 4.762 | **<0.001** | 0.997 | 0.467 | 2.133 | **0.033** | 0.643 | 0.274 | 2.345 | **0.019** | -0.776 | 0.457 | -1.698 | 0.089 |  |
| Moved | 0.821 | 0.315 | 2.605 | **0.009** | 0.091 | 0.002 | 41.42 | **<0.001** | 0.382 | 0.252 | 1.513 | 0.13 | -0.943 | 0.477 | -1.976 | 0.048 |  |
| Pride | 0.237 | 0.252 | 0.94 | 0.347 | 0.153 | 0.311 | 0.491 | 0.624 | 0.656 | 0.222 | 2.961 | **0.003** | 0.503 | 0.180 | 2.792 | **0.005** |  |
| Relief | 4.274 | 0.548 | 7.795 | **<0.001** | 0.830 | 0.326 | 2.548 | **0.012** | 2.924 | 0.338 | 8.641 | **<0.001** | 1.227 | 0.263 | 4.658 | **<0.001** |  |
| Respected | 0.60 | 0.284 | 0.916 | 0.36 | 0.771 | 0.247 | 3.124 | **0.002** | -0.816 | 0.690 | -1.183 | 0.237 | -0.300 | 0.333 | -0.899 | 0.369 |  |
| Schadenfreude | 1.642 | 0.269 | 6.1 | **<0.001** | -0.121 | 0.441 | -0.275 | 0.784 | 2.494 | 0.282 | 8.841 | **<0.001** | -0.070 | 0.351 | -0.2 | -0.841 |  |
| Sensory Pleasure | 1.642 | 0.269 | 6,1 | **<0.001** | -0.366 | 0.383 | -0.956 | 0.339 | 1.408 | 0.177 | 7.976 | **<0.001** | -0.090 | 0.003 | -31.11 | <0.001 |  |
| Surprise | 1.671 | 0.187 | 8.933 | **<0.001** | 1.319 | 0.169 | 7.752 | **<0.001** | 1.635 | 0.300 | 5.456 | **<0.001** | -0.076 | 0.363 | -0.211 | 0.833 |  |
| Tenderness | 2.194 | 0.339 | 6.472 | **<0.001** | 0.208 | 0.249 | 0.833 | 0.405 | 0.049 | 0.352 | 0.14 | 0.888 | 0.570 | 0.291 | 1.956 | 0.051 |  |
| Triumph | 0.284 | 0.313 | 0.907 | 0.365 | 0.626 | 0.254 | 2.462 | **0.012** | 1.613 | 0.330 | 4.889 | **<0.001** | 0.479 | 0.200 | 2.398 | **0.017*** |  |

*Note.* Bold mark indicates performance accuracy better than expected by chance.

**Table 2S.** Random effects in GLMM models comparing recognition accuracy to chance level per emotion

| Random effects | **Dutch nonverbal vocalizations** | | **Dutch speech prosody** | | **Chinese nonverbal vocalizations** | | | **Chinese speech prosody** | | | |
| --- | --- | --- | --- | --- | --- | --- | --- | --- | --- | --- | --- |
|  | **ParticipantID (Variance,*SD*)** | **VocalizationID (Variance,*SD*)** | **ParticipantID (Variance,*SD*)** | **VocalizationID (Variance,*SD*)** | **ParticipantID (Variance,*SD*)** | **VocalizationID (Variance,*SD*)** | | **ParticipantID (Variance,*SD*)** | **VocalizationID (Variance,*SD*)** | | |
| Admiration | 1.627,1.275 | 1.480,1.217 | 0.277,0.526 | 0.482,0.695 | 0.823,0.907 | | 0.701,0.837 | 0.000,0.000 | | 0.208,0.456 | |
| Amae | 1.277,1.130 | 0.206,0.454 | 0.288,0.536 | 0.647,0.805 | 1.601,1.265 | | 2.379,1.543 | 0.000,0.000 | | 2.153,1.467 | |
| Amusement | 0.987,0.993 | 2.092,1.446 | 0.335,0.579 | 3.105,1.762 | 1.509,1.228 | | 0.575,0.758 | 0.098,0.314 | | 1.119,1.058 |  |
| Awe | 1.665,1.290 | 1.273,1.128 | 0.804,0.897 | 0.004,0.060 | 0.151,0.38 | | 0.278,0.527 | 0.167,0.408 | | 0.041,0.202 |  |
| Determination | 0.000,0.000 | 0.258,0.501 | 0.000,0.000 | 0.456,0.675 | 0.316,0.562 | | 1.604,1.266 | 0.683,0.826 | | 0.617,0.785 |  |
| Elation | 0.000,0.000 | 0.000,0.000 | 0.000,0.000 | 0.456,0.675 | 0.000,0.000 | | 0.846,0.919 | 0.503,0.709 | | 0.674,0.821 |  |
| Elevation | 0.000,0.0000 | 0.000,0.0000 | 0.000,0.0000 | 0.146,0.382 | 0.001,0.037 | | 0.612,0.782 | 0.165,0.406 | | 0.206,0.453 |  |
| Excitement | 0.070,0.264 | 1.249,1.118 | 0.201,0.448 | 4.812,2.194 | 0.852,0.923 | | 0.428,0.654 | 0.000,0.000 | | 0.000,0.000 |  |
| Gratitude | 0.000,0.000 | 0.675,0.822 | 0.766,0.875 | 0.258,0.508 | 2.162,1.471 | | 0.000,0.000 | 1.441,1.201 | | 0.000,0.000 |  |
| Hope | 0.000,0.000 | 1.108,1.052 | 0.700, 0.837 | 0.525,0.725 | 2.629,1.621 | | 0.518,0.720 | 0.000,0.000 | | 0.000,0.000 |  |
| Inspiration | 0.397,0.630 | 0.590,0.768 | 0.509,0.713 | 0.656,0.810 | 0.275,0.525 | | 0.487,0.698 | 0.000,0.000 | | 0.383,0.619 |  |
| Interest | 0.000,0.000 | 0.834,0.913 | 0.000,0.000 | 0.498,0.705 | 0.747,0.864 | | 0.866,0.930 | 0.000,0.000 | | 0.000,0.000 |  |
| Lust | 0.494,0.703 | 2.201,1.484 | 0.309,0.556 | 3.492,1.869 | 0.805,0.897 | | 0.516,0.719 | 0.823,0.907 | | 0.146,0.382 |  |
| Moved | 0.000,0.000 | 1.454,1.206 | 0.000,0.000 | 0.790,0.889 | 0.759,0.871 | | 0.121,0.348 | 1.208,1.099 | | 0.000,0.000 |  |
| Pride | 0.332,0.576 | 0.227,0.477 | 0.417,0.646 | 0.630,0.794 | 0.082,0.286 | | 0.319,0.565 | 0.000,0.000 | | 0.000,0.000 |  |
| Relief | 3.898,1.974 | 1.743,1.320 | 0.011,0.107 | 1.474,1.214 | 0.162,0.403 | | 1.700,1.304 | 0.014,0.120 | | 0.946,0.973 |  |
| Respected | 0.099,0.315 | 0.567,0.753 | 0.000,0.000 | 0.000,0.000 | 4.894,2.212 | | 1.738,1.318 | 1.108,1.052 | | 0.074,0.272 |  |
| Schadenfreude | 0.478,0.691 | 0.954,0.977 | 0.635,0.797 | 1.839,1.356 | 0.737,0.858 | | 1.012,1.006 | 0.228,0.478 | | 0.902,0.950 |  |
| Sensory Pleasure | 0.478,0.691 | 0.954,0.977 | 0.438,0.661 | 0.531,0.729 | 0.138,0.3712 | | 0.2467,0.497 | 0.000,0.000 | | 0.373,0.611 |  |
| Positive Surprise | 0.000,0.000 | 0.385,0.621 | 0.037,0.192 | 0.198,0.445 | 0.164,0.405 | | 1.356,1.164 | 0.000,0.000 | | 1.372,1.171 |  |
| Tenderness | 1.160,1.077 | 1.588,1.260 | 0.000,0.000 | 0.509.0.713 | 0.393,0.627 | | 0.893,0.945 | 0.089,0.298 | | 0.937,0.968 |  |
| Triumph | 0.694,0.833 | 0.751,0.867 | 0.128,0.357 | 0.569,0.754 | 0.177,0.421 | | 1.709,1.307 | 0.000,0.000 | | 0.282,0.531 |  |

**Table 3S.** *Random effects in GLMM models comparing recognition accuracy across vocalization types*

| Random effects | **Dutch** | | | **Chinese** | |
| --- | --- | --- | --- | --- | --- |
|  | **ParticipantID (Variance,*SD*)** | | **VocalizationID (Variance,*SD*)** | **ParticipantID (Variance,*SD*)** | **VocalizationID (Variance,*SD*)** |
| Admiration | 0.446,0.668 | | 0.152,0.389 | 0.342,0.585 | 0.429,0.655 |
| Amae | 0.435,0.660 | | 0.505,0.711 | 0.503,0.709 | 1.409,1.187 |
| Amusement | 0.391,0.626 | | 1.558,1.248 | 0.715,0.846 | 0.802,0.895 |
| Awe | 1.049,1.024 | | 0.338,0.581 | 0.143,0.379 | 0.127,0.356 |
| Determination | 0.000,0.000 | | 0.000,0.000 | 0.428,0.654 | 0.433,0.658 |
| Elation | 0.140,0.374 | | 1.105,1.051 | 0.107,0.327 | 0.610,0.788 |
| Elevation | 0.000,0.000 | | 0.000,0.0000 | 0.015,0.122 | 0.238,0.488 |
| Excitement | 0.063,0.252 | | 1.608,1.268 | 0.324,0.569 | 0.123,0.350 |
| Gratitude | 0.045,0.212 | | 0.333,0.577 | 1.925, 1.388 | 0.000,0.001 |
| Hope | 0.149,0.386 | | 0.400,0.633 | 0.585,0.765 | 0.030,0.174 |
| Inspiration | 0.454,0.674 | | 0.716,0.846 | 0.156,0.394 | 0.469,0.685 |
| Interest | 0.000,0.000 | | 0.000,0.000 | 0.261,0.510 | 0.401,0.634 |
| Lust | 0.176,0.419 | | 1.207,1.099 | 0.699,0.836 | 0.260,0.510 |
| Moved | 0.000,0.000 | | 0.776,0.881 | 0.837,0.915 | 0.010,0.101 |
| Pride | 0.355,0.596 | | 0.303,0.551 | 0.000,0.000 | 0.000, 0.479 |
| Relief | 1.069,1.034 | | 0.957,0.978 | 0.091,0.301 | 1.234,1.111 |
| Respected | 0.000,0.000 | | 0.000,0.000 | 1.849,1.360 | 0.262,0.512 |
| Schadenfreude | 0.452,0.672 | 0.580,0.762 | | 0.510,0.714 | 0.212,0.460 |
| Sensory Pleasure | 0.308,0.555 | 1.036,1.018 | | 0.062,0.251 | 0.006,0.080 |
| Surprise | 0.000,0.000 | | 0.000,0.000 | 0.083,0.289 | 0.979,0.990 |
| Tenderness | 0.311,0.558 | | 0.552,0.743 | 0.053,0.231 | 0.387,0.622 |
| Triumph | 0.371,0.609 | | 0.314,0.560 | 0.000,0.000 | 0.845,0.919 |
